# Description of subgroup reporting in clinical trials of chronic diseases: a meta-epidemiological study

**DOI:** 10.1136/bmjopen-2023-081315

**Published:** 2024-06-20

**Authors:** Lili Wei, Elaine Butterly, Jesús Rodríguez Pérez, Avirup Chowdhury, Richard Shemilt, Peter Hanlon, David McAllister

**Affiliations:** 1 University of Glasgow School of Health and Wellbeing, Glasgow, UK; 2 Institute of Cancer Research, London, UK

**Keywords:** randomized controlled trial, chronic disease, epidemiology

## Abstract

**Abstract:**

**Introduction:**

In trials, subgroup analyses are used to examine whether treatment effects differ by important patient characteristics. However, which subgroups are most commonly reported has not been comprehensively described.

**Design and settings:**

Using a set of trials identified from the US clinical trials register (ClinicalTrials.gov), we describe every reported subgroup for a range of conditions and drug classes.

**Methods:**

We obtained trial characteristics from ClinicalTrials.gov via the Aggregate Analysis of ClinicalTrials.gov database. We subsequently obtained all corresponding PubMed-indexed papers and screened these for subgroup reporting. Tables and text for reported subgroups were extracted and standardised using Medical Subject Headings and WHO Anatomical Therapeutic Chemical codes. Via logistic and Poisson regression models we identified independent predictors of result reporting (any vs none) and subgroup reporting (any vs none and counts). We then summarised subgroup reporting by index condition and presented all subgroups for all trials via a web-based interactive heatmap (https://ihwph-hehta.shinyapps.io/subgroup_reporting_app/).

**Results:**

Among 2235 eligible trials, 23% (524 trials) reported subgroups. Follow-up time (OR, 95%CI: 1.13, 1.04–1.24), enrolment (per 10-fold increment, 3.48, 2.25–5.47), trial starting year (1.07, 1.03–1.11) and specific index conditions (eg, hypercholesterolaemia, hypertension, taking asthma as the reference, OR ranged from 0.15 to 10.44), predicted reporting, sponsoring source and number of arms did not. Results were similar on modelling any result reporting (except number of arms, 1.42, 1.15–1.74) and the total number of subgroups. Age (51%), gender (45%), racial group (28%) were the most frequently reported subgroups. Characteristics related to the index condition (severity/duration/types etc) were frequently reported (eg, 69% of myocardial infarction trials reported on its severity/duration/types). However, reporting on comorbidity/frailty (five trials) and mental health (four trials) was rare.

**Conclusion:**

Other than age, sex, race ethnicity or geographic location and characteristics related to the index condition, information on variation in treatment effects is sparse.

**PROSPERO registration number:**

CRD42018048202.

**STRENGTHS AND LIMITATIONS OF THIS STUDY**

- The assessment of subgroup reporting was not restricted to major journals.
- We assigned a standard terminology to every subgroup, rather than using a restricted list.
- All trials had to be registered on ClinicalTrials.gov, and all corresponding papers had to be notified to ClinicalTrials.gov or indexed in PubMed with a ClinicalTrials.gov ID.
- A small number of subgroup terms remained unassigned to standard terminologies due to their complexity.

## Introduction

 Subgroup analyses in randomised clinical trials (hereafter trials) are used to examine consistency/differences in treatment effects between groups to help tailor treatment recommendations and provide reassurance that treatment effects are ‘portable’ to groups with different characteristics.^1^ However, individual trials are rarely sufficiently large to estimate subgroup effects with adequate precision, making subgroup effect estimates difficult to interpret and frequently misleading.^2 3^

To help address this problem, subgroup analyses of similar trials can be combined in meta-analyses.^4^ However, this requires that the subgroups of interest are reported consistently across multiple trials.^4^ A number of studies have examined the reporting of subgroups but have mostly focused on subgroup reporting as a whole (eg, the incidence and determinants of subgroup reporting, and the extent to which reporting conforms to guidelines),^5^,^8^ rather than on which subgroups are most commonly reported. Also the focus has mostly been on individual papers—particularly papers in major medical journals—rather than on total trial subgroup reporting. This means that some trial subgroup reporting may be missed.

As such, while subgroup reporting is inconsistent overall, it remains unclear for specific index conditions and types of intervention, which subgroups are most and least consistently reported. Such information would help those planning systematic reviews. Alongside other important considerations such as clinical factors, biological plausibility and statistical constraints, this could also inform the development of a standard set subgroups for different index conditions and interventions.

Therefore, using trials we previously identified from the US clinical trials register (ClinicalTrials.gov), we describe every reported subgroup across a wide range of conditions and drug classes. Using standard terminologies, we have described and categorised all reported subgroups and summarised these according to trial index conditions.

## Methods

### Identifying trials registered on ClinicalTrials.gov

The trial selection has been described previously^9^. Briefly, we searched on 4 September 2017 for trials of pharmacological treatments for medical disorders registered on clincialtrials.gov between January 1990 and November 2016 using the Access to Aggregate Content of ClinicalTrials.gov (AACT) database, which is a complete copy of ClinicalTrials.gov in a relational database format.^10^ The selection criteria include phase 2/3, 3 or 4 trials, recruiting ≥300 participants, with an upper age limit of ≥60 years or no maximum^9^ (online supplemental table 1). Conditions were chosen based on the requirement for long-term pharmacological therapy, including a range of cardiovascular, musculoskeletal, gastrointestinal, respiratory, neurological, urological, metabolic and autoimmune disorders. A full list of included conditions, Medical Subject Heading (MeSH) terms and MeSH code are provided in online supplemental table 2.

### Identifying publications relating to registered trials

We searched for all PubMed-indexed publications relating to any trials identified from ClinicalTrials.gov. First, we searched ClinicalTrials.gov for PubMed IDs (PMIDs) of all relevant registered trials. Trial sponsors are required to update the ClinicalTrials.gov database with PMIDs of publications related to registered trials. Second, to identify publications which had not been added to the database, we searched PubMed using the trial registration number for each relevant trial. This search was performed using the R Eutils package.^11^ This was last updated in April 2019.

### Screening of publications

**Figure 1:**
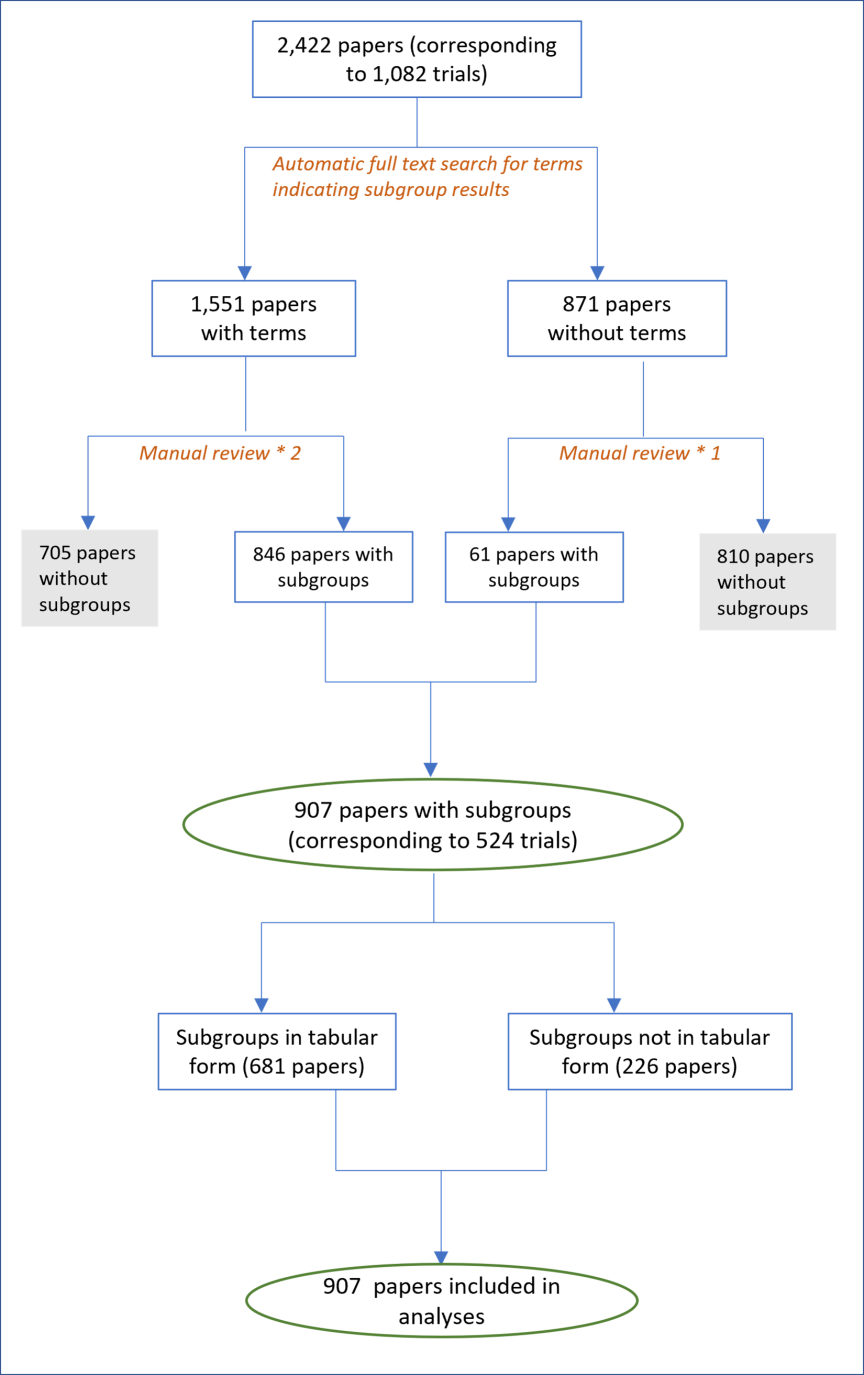
Screening of subgroup analyses from papers reporting any overall trial results.

We screened all papers manually and via automatic text searches as shown in figure 1. First, an automatic full-text search was performed using the following strings “subgroup”, “sub-group” “strata”, “by baseline”, “subpopulation”, or “sub-population”. Where automatic screening did not identify any of these terms in the manuscript text, articles (including appendices) were manually screened once to check the true negative results, otherwise studies were screened two times by two independent reviewers.

### Data extraction

Trial-level data for all trials identified from ClinicalTrials.gov, regardless of publication status and the presence of subgroup analyses, were extracted from AACT. Extracted data included ClinicalTrials.gov identifier, index condition, interventions and comparators, number of participants, phase of trial, number of arms, trial sponsor, start date, completion date, countries included and eligibility criteria. Subgroup data were extracted exclusively from publications. In our experience, subgroup results are rarely added to clincialtrials.gov. Tabulated subgroup data were extracted using an interactive web-app TableTidier that we developed in house (https://tabletidier.org/), subgroup results in the manuscript text or in figures without accompanying tables were extracted manually. To allow comparison of subgroups across different studies, extracted subgroups were assigned to standard terms using the MeSH and/or WHO Anatomical Therapeutic Chemical (ATC) vocabularies. MeSH is created by National Library of Medicine (NLM) for indexing journal articles and books, which is widely used by PubMed and ClinicalTrials.gov. Initial assignments were made within TableTidier. All assignments were then reviewed by a clinically qualified investigator and given a final designation. Some additional qualifiers were added to assigned MeSH terms for subgroups such as disease severity or duration to capture more information (ie, duration of diabetes is one of the subgroups in diabetes trials).

For index conditions, we used the original MeSH terms assigned when trials were registered. Briefly, when registering a study, data submitters are required to provide condition using MeSH terms. Furthermore, an NLM algorithm assesses submitted text and assigns MeSH terms. More details of this process are available in section 2.1 ‘Use of MeSH Terminology in the ClinicalTrials.gov Database’.^12^

### Statistical analysis

Via an interactive heatmap, we summarised all original subgroup terms and MeSH terms at the level of individual subgroup for all trials. The heatmap allows users to examine subgroup reporting according to the type of subgroup, index condition, drug class and other trial characteristics (https://ihwph-hehta.shinyapps.io/subgroup_reporting_app/), and where possible, links directly to the extracted tables (a video demonstration is available in the online supplemental file 3). For this manuscript, to provide a concise overview, we present simple summary statistics such as ranks, counts and percentages and also present particular terms of interest and terms collapsed into broader categories using the MeSH hierarchy (eg, we collapsed heart failure and myocardial infarction into cardiovascular diseases (CVDs)).^13^

We fitted two sets of logistic regression models for (1) any overall results reported (if trials reported trial results at all) and (2) any subgroups reported (taking those with any overall results reported as the denominator). For both outcomes, multivariate regression models were used. Variables included were the year the trial started, number of arms ((>2 arms vs ≤2 arms), number of participants enrolled (log-transformed with a base of 10, so that the coefficient corresponds to the increase in overall results reporting or subgroup reporting per 10-fold increment in sample size), sponsor type (industry vs other), duration of follow-up and the index condition (table 1 and online supplemental table 3). The coefficients were presented on the exponential scale. Among trials with any subgroup reporting, we used quasi-Poisson models to examine the total number of subgroups. ‘One subgroup’ indicates whether there are multiple levels (eg, ‘age’ includes both <65 and >65-year-olds, ‘sex’ includes both women and men), we count each subgroup only once. For the quasi-Poisson model, the outcome was the count of subgroups per trial, and covariates included were the same as those in the regression models. Data analysis was performed using R V.4.2. Data and selected code are available at GitHub repository (https://github.com/ChronicDiseaseEpi/subgroup_reporting.git).

**Table 1:** The proportion of subgroup reporting and the most common subgroups in each index condition

Conditions	Total subgroups	The proportion of subgroup reporting among 2235 trials n_T_/N (%)	The proportion of subgroup reporting among 1082 trials with results reporting n_R_/N_R_ (%)	Five the most common subgroups in each condition
Myocardial infarction	99	26/47 (55)	25/30 (83)	Age factors (96.2%); diabetes mellitus (88.5%); gender identity (88.5%); **myocardial infarction (69.2%**); hypertension (30.8%)
Diabetes mellitus, type 2	89	120/460 (26)	117/235 (50)	Age factors (49.17%); glycated haemoglobin a (48.33%); gender identity (39.17%); body mass index (36.67%); racial groups (36.67%)
Coronary artery disease	77	27/80 (34)	27/46 (59)	Diabetes mellitus (85.2%); age factors (74.1%); gender identity (74.1%); myocardial infarction (37.0%); hypertension (33.3%)
Hypertension	64	44/247 (18)	44/98 (45)	Age factors (59.1%); gender identity (52.3%); diabetes mellitus (38.6%); racial groups (36.4%); blood pressure (27.3%)
Heart failure	51	17/40 (42)	17/27 (63)	Age factors (70.6%); diabetes mellitus (64.7%); gender identity (64.7%); stroke volume (58.8%); **heart failure (52.9%**)
Hypercholesterolaemia	48	28/72 (39)	28/43 (65)	Lipoproteins (71.4%); diabetes mellitus (67.9%); age factors (64.3%); gender identity (60.7%); body mass index (53.6%)
Atrial fibrillation	46	13/39 (33)	13/20 (65)	Age factors (61.5%); gender identity (53.8%); heart failure (53.8%); **atrial fibrillation (46.2%**); hypertension (38.5%)
Pulmonary disease, chronic obstructive	40	40/186 (22)	39/96 (41)	**Pulmonary disease, chronic obstructive (75.0%**); age factors (50.0%); cigarette smoking (45.0%); gender identity (42.5%); steroids (40.0%)
Acute coronary syndrome	37	9/22 (41)	9/10 (90)	Age factors (89%); gender identity (78%); diabetes mellitus (67%); myocardial infarction (56%); percutaneous coronary intervention (56%)
Arthritis, rheumatoid	35	28/106 (26)	28/65 (43)	**Arthritis, rheumatoid (46.4%**); age factors (25.0%); gender identity (21.4%); immunosuppressive agents (21.4%); c reactive protein (17.9%)
Stroke	35	8/20 (40)	8/13 (62)	**Stroke (88%**); age factors (62%); gender identity (62%); diabetes mellitus (38%); hypertension (38%)
Atherosclerosis	30	2/9 (22)	2/3 (67)	Age factors (100%); body mass index (100%); cigarette smoking (100%); diabetes mellitus (100%); gender identity (100%)
Crohn disease	29	11/18 (61)	11/16 (69)	Immunosuppressive agents (63.6%); tumour necrosis factor inhibitors (63.6%); c reactive protein (54.5%); **Crohn disease (45.5%**); steroids (45.5%)
Osteoporosis	29	11/44 (25)	11/23 (48)	Age factors (54.5%); fractures, bone (54.5%); **osteoporosis (45.5%**); body mass index (27.3%); geographic locations (27.3%)
Prostatic hyperplasia	28	9/30 (30)	9/15 (60)	Body mass index (44%); age factors (33%); erectile dysfunction (33%); adrenergic alpha-antagonists (22%); antihypertensive agents (22%)
Peripheral arterial disease	24	3/8 (38)	3/4 (75)	Diabetes mellitus (67%); age factors (33%); ankle brachial index (33%); blood pressure (33%); body weight (33%)
Venous thromboembolism	23	7/36 (19)	7/8 (88)	Age factors (86%); gender identity (86%); **venous thromboembolism (57%**); anticoagulants (43%); body weight (43%)
Asthma	22	19/147 (13)	19/62 (31)	**Asthma (31.6%**); eosinophilia (31.6%); steroids (26.3%); age factors (21.1%); gender identity (21.1%)
Colitis, ulcerative	21	8/14 (57)	8/12 (67)	Steroids (62%); tumour necrosis factor inhibitors (62%); c reactive protein (38%); gender identity (38%); age factors (25%)
Psoriasis	19	13/62 (21)	13/37 (35)	Immunosuppressive agents (38.5%); **psoriasis (38.5%**); tumour necrosis factor inhibitors (30.8%); biological therapy (15.4%); cyclosporins (15.4%)

This table is ordered by the total number of subgroups in each index condition, and only the first 20 rows are displayed. The entire table is available in the appendix (Supplementary Table 3online supplemental table 3). Some trials might correspond to multiple index conditions, we kept the commonestmost common condition among 2235 trials for simplicity; the number for some subgroups is the same in the 5thfifth place and only one was kept based on the alphabetical order; the subgroup in bold is the subgroup same as the condition term with additional information such as type, severity, duration, etc; n_T:_ number of trials with subgroup reporting among 2235 trials; n_R:_ number of trials with subgroup reporting among 1082 trials with results reporting; N_R:_ trials with results reporting and N_R_=1082.

### Differences with other studies citing the same PROSPERO registration

Previous studies using the same PROSPERO registration differed with this study in data usage and research questions. Among trials identified from this PROSPERO registration, papers by Lees *et al* and Butterly *et al* used a subset trials with individual participant data (IPD); Lees *et al* estimated the association between participant characteristics (age, sex and morbidity counts) and trial attrition^14^ and Butterly *et al* examined associations between comorbidity count on quality of life.^15^ In the current study, we used all trials whether or not IPD was available, examining subgroups from papers and trial-level data from AACT database.

### Patient and public involvement

None.

## Results

As reported previously,^9^ we identified 2235 registered trials with a prespecified set of conditions and treatment comparisons. Among these, 1082 trials reported overall published results (online supplemental figure S1), with 524 (48.43%) trials reporting findings from subgroup analyses (figure 1). We reduced over 2000 unique strings to 345 MeSH terms and 182 were further described using qualifiers (eg, severity, duration).

### Presence and number of subgroups reported

Of the 524 trials reporting subgroups, 156 (30%) reported a single subgroup, 90 (17%) reported 2–3 subgroups, 73 (14%) reported 4–5 subgroups and 205 (39%) reported six or more subgroups. Compared with trials without subgroup reporting, trials reporting subgroups were generally larger (median and IQR: 827 participants, 499–1912) vs 610 (418–1000), had longer follow-up (years, 2, 2–4) vs 2 (1-3), a higher percentage of non-industry sponsorship (14% vs 9%) and a higher percentage with more than two arms (39% vs 35%).

**Figure 2:**
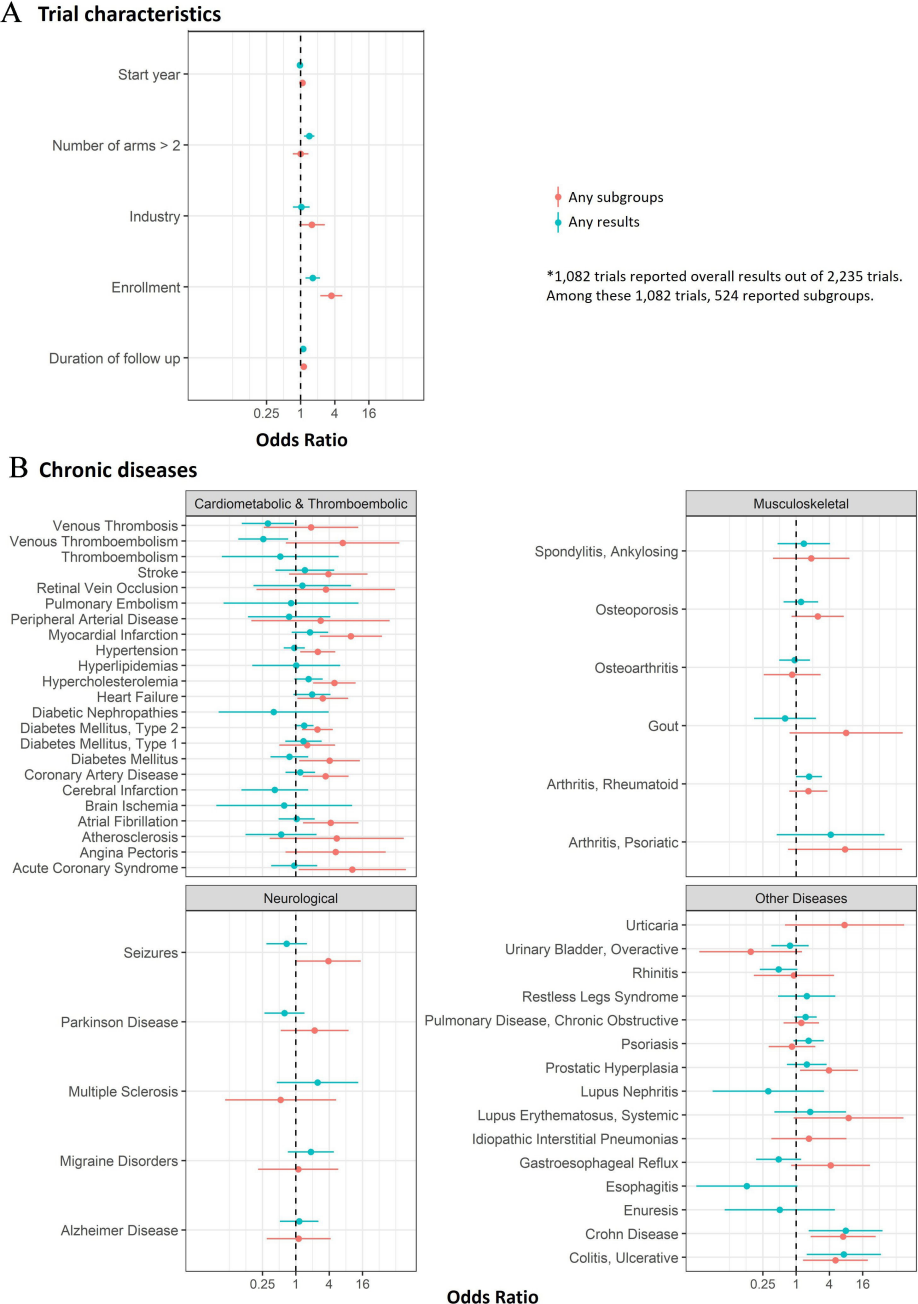
The associations between (A) trial characteristics, (B) chronic diseases and subgroup reporting and overall results reporting, respectively.

Figure 2 shows associations for any overall result reporting (yes/no among 2235 trials) and any subgroup reporting (yes/no among 1082 trials reporting overall results). Of the trial characteristics (figure 2A), the number of participants enrolled was the most important predictor of any overall result reporting (OR and 95% CI per 10-fold increase in number enrolled (eg, from 10 to 100): 1.63, 1.22–2.19), and any subgroup reporting (3.48, 2.25–5.47) and the total number of subgroups reported (see online supplemental table 4.1: rate ratio (RR) per 10-fold increase 1.69, 1.65–1.73). Duration of follow-up also predicted any result reporting (OR per year increase in follow-up 1.10, 1.03–1.18), subgroup reporting (1.13, 1.04–1.24) and the total number of subgroups (RR 1.03, 1.02–1.03). More recent trials were similar to older trials (OR 0.97, 0.95–0.99; OR 1.07, 1.03–1.11 and RR 1.02, 1.02–1.02, respectively). Trials with three or more arms were more likely to report results (OR 1.42, 1.15–1.74) but were not associated with increased subgroup reporting (OR 1.00, 0.73–1.37) or a higher total number of subgroups (RR 1.01, 0.99–1.04). Industry sponsoring was not associated with any of the three outcomes (OR 1.03, 0.73–1.45; OR 1.58, 0.94–2.69 and RR 1.00, 0.97–1.03, respectively).

Taking asthma trials as a reference (as it has lower odds of reporting to make the ratios easier to interpret), subgroup reporting was more likely within trials of cardiovascular, metabolic, thromboembolic index conditions (figure 2B and online supplemental table 4.2, overall index conditions ORs ranged from 0.15 to 10.44). These trials were also more likely to report larger numbers of subgroups (online supplemental table 4.1). Results for other indications were more mixed.

### The most common subgroups reported

There was substantial variation in subgroups across index conditions. Across 49 index conditions, there were a total of 345 subgroup terms, with a median of 11 subgroup terms per index condition ranging from 1 to 99 (IQR 6 to 29). Nonetheless, some subgroups were common across all index conditions. Age (268 out of 524 trials, 51%) and gender (235 trials, 45%) were the most common, followed comorbid diabetes (154 trials, 29%), racial group (146 trials, 28%), body mass index (BMI) (125 trials, 24%), geographical locations (88 trials, 17%), Glycated haemoglobin A (72 trials, 14%) and cigarette smoking (63 trials, 12%). Most of the BMI subgroup appeared in type 2 diabetes mellitus (T2DM) trials (n=44, out of 125 trials reporting BMI, 35%). Most of the cigarette smoking subgroups were in chronic obstructive pulmonary disease (COPD) trials (n=18, out 63 trials reporting smoking, 29%), followed by coronary artery disease (13%, n=8) and T2DM trials (11%, n=7).

For many trials, subgroups relating to the index condition (eg, duration or severity) were commonly reported which meant treatment effects were stratified by the type, duration or severity of the index condition. For example, among 26 myocardial infarction trials with subgroup analyses, 69% reported severity/history/type of myocardial infarction as a subgroup, for T2DM trials, 29 of 120 trials reported diabetes characteristics (mainly duration) as a subgroup and for COPD, 30 of 40 trials (75%) reported severity of COPD as a subgroup, while 88% stroke trials reported previous/severity/type of stroke as a subgroup (table 1).

### Comorbidity subgroup reporting

**Figure 3:**
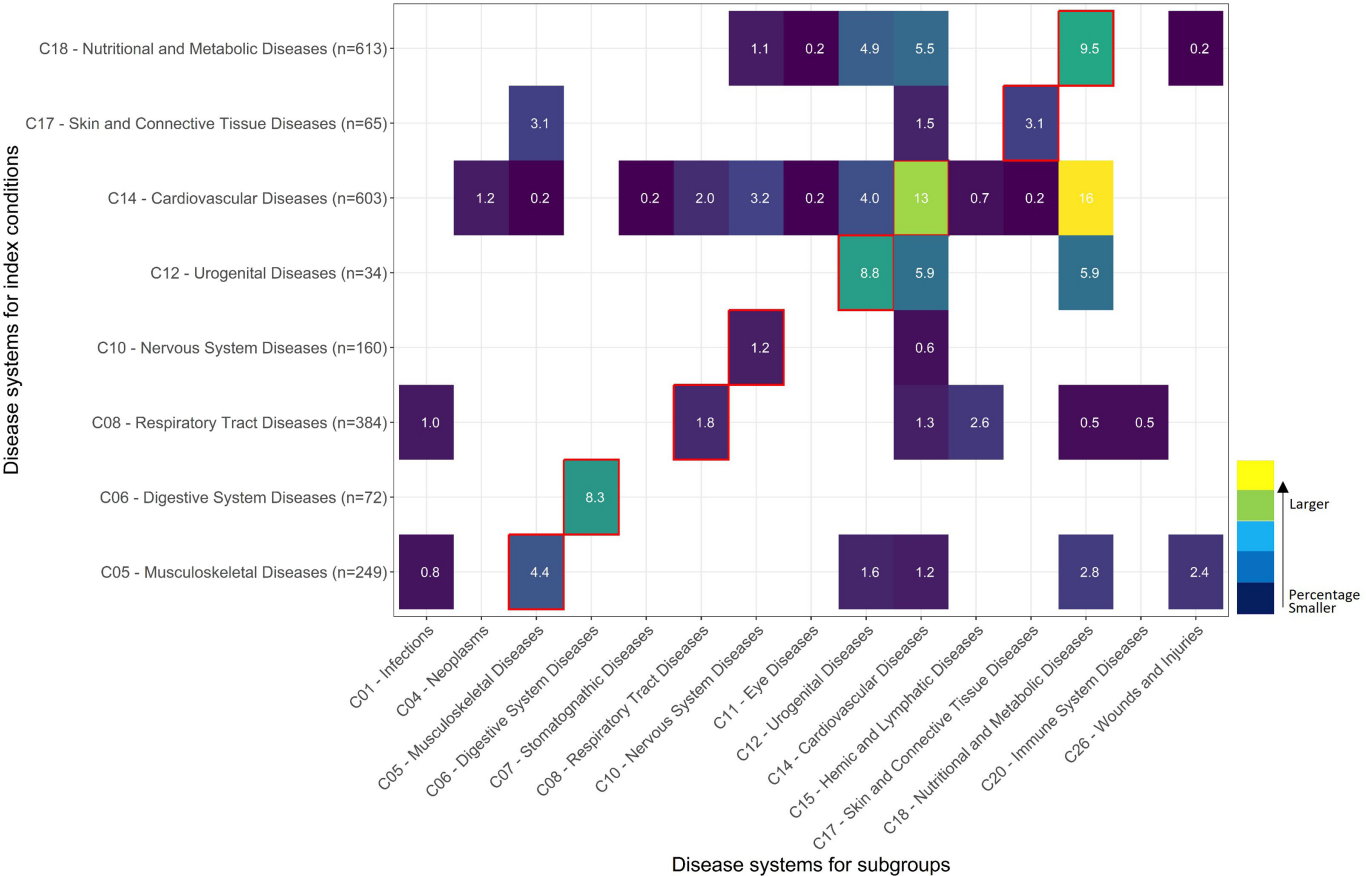
Comorbidity subgroup reporting.

Where conditions other than the same index conditions were reported as subgroups, this was largely confined to conditions within the same body system. Figure 3 illustrates this—the organ system for each index condition and each subgroup are shown on the y and x-axis, respectively, and the % of subgroups reported per organ system are shown on each cell. Where the index condition and subgroup pertain to the same organ system, the cells are outlined in red. Otherwise, if they are in different organ systems, the cells are not bordered. Frequencies above 5% were generally seen on the cells with red borders (eg, 13% CVD trials reported a non-index condition CVD subgroup—eg, stroke trials reported hypertension as a subgroup which are both CVDs). Where there were high percentages not in red borders, the subgroup conditions were either known causes or known sequelae of the index condition such as nutritional and metabolic disease (predominantly diabetes) in CVD trials (16%) or CVDs (5.5%) and renal disease (4.9% urogenital diseases) in diabetes trials. In contrast, only 1.3% of respiratory tract disease trials reported subgroup results according to presence/characteristics of CVDs.

### Comorbidity, multimorbidity, frailty and mental health

Trials rarely included metrics of comorbidity, multimorbidity or frailty (five trials). 78 trials (15%) reported estimated glomerular filtration rate or renal insufficiency as renal impairment measures and the majority were either T2DM trials (n=28) or heart failure trials (n=8). Subgroups related to mental health were particularly rarely reported with only four trials (1%).

## Discussion

On reviewing 2235 trials registered on ClinicalTrials.gov, we made a number of observations about subgroup reporting. First, only around a quarter of trials report subgroup effects. Second, of those reporting subgroup effects, just under half (47%) report on three or fewer subgroups. Third, the sample size, duration of trial follow-up and trial starting year predict subgroup reporting. Fourth, after accounting for participants enrolled, industry-sponsored trials are not more likely to report subgroup effects. Fifthly, some trials with conditions of cardiovascular, metabolic and thromboembolic disease are the most likely to report subgroups.

Finally, we showed that even where trials do report subgroups, this is largely confined to ‘general’ subgroups such as age, sex, race/ethnicity, geographic variation or to features of the index condition. Mental health disorders or metrics of comorbidity, multimorbidity or frailty were rarely reported. Together these findings suggest that—with the exception of cardiometabolic and thromboembolic diseases, and especially for subgroups not closely related to the index condition—the published literature we screened contains only sparse information on how treatment effects differ within trials.

### Strengths and weaknesses of our study

A strength of our study is that, unlike most previous studies,^16 17^ we included registered trials regardless of where they were published. Second, our study was the largest of which we are aware to assess subgroup reporting among trials of chronic medical conditions. Third, this was the only study to assign terms to standard terminologies allowing comparison across multiple conditions and drug classes. However, there were some limitations. First, where papers were neither notified to ClinicalTrials.gov nor included a trial registration identifier in PubMed, we will not have obtained the relevant study. However, this number missed is likely to be small because the trial registration number is required by the International Committee of Medical Journal Editors.^18^ Second, subgroup results in non-indexed sources (eg, clinical study reports) will have been missed, although many are only accessible after a formal application. Third, a small number of terms could not be assigned to MeSH or ATC codes due to complexity. Finally, our results are confined to chronic diseases and exclude trials in infectious diseases, oncology and psychiatric disorders (other than dementia).

### Strengths and weaknesses in relation to other studies

Many previous subgroup studies were concerned with the reliability of subgroup findings in the context of a single paper. As such, since higher impact journals are likely to be more influential, most confined their analysis to papers published with them.^5 6^ One study examined all papers regardless of the journal but was confined to trials with protocols approved 10 or more years ago.^19^ This difference in papers included could account for the fact that previous studies found an association between industry sponsoring and subgroup reporting while we did not, as the denominator they used was trials with protocols. Taji *et al* also showed that trial protocols with subgroup planning were more likely to be industry sponsored than those without planned subgroup.^20^ Alternatively, our null association for industry funding could be due to the unmeasured confounding—that is, unmeasured differences between industry and non-industry trials, which are related to subgroup reporting.

Nevertheless, our study and previous research share a number of common findings, particularly larger studies were more likely to report subgroup effects.^6 21^ One study reported detailed information on *which* subgroups were reported,^6^ categorising 1042 subgroups into demographics (25%), comorbidities (10%), disease severity (32%) and more. Some were further subcategorised. For example, comorbidity was categorised into diabetes (31%), CVD (35%) and demographics into age, sex, race/ethnicity. These percentages appear consistent with our observations as to which subgroups were most common, although treating the variables examined as the denominator meant that it cannot be directly compared with our findings. Even in examining trial protocols, age and sex are the most frequently planned subgroups.^20^

### Meaning of the study

According to the Cochrane Handbook for Systematic Reviews of Interventions, subgroup analyses are ‘uncommon in systematic reviews based on published literature because sufficient details to extract data about separate participant types are seldom published’.^22^ We found that considerable variation in reporting between trials even within the same index condition and drug class was one reason for this lack of detail. Nonetheless, common variables did emerge such as age, sex, race/ethnicity and features of the index condition.

In contrast, we found there was very little information about comorbidity and multimorbidity. Given that multimorbidity is common, increasing in prevalence, and is known to complicate clinical decision-making, the lack of such information is a challenge for decision-makers.^23^ We previously showed that, while under-represented, multimorbidity is not absent from trials.^9^ Despite this, very few trials have reported treatment effects according to comorbidity, multimorbidity or frailty. Moreover, for individual comorbidities, the majority of reporting was for conditions in the same body system as the index disease (eg, history of ischaemic heart disease in antihypertensive trials), so there was little information about ‘discordant’ comorbidities (eg, coexisting prostate disease and heart failure), which are the most complex and difficult to treat. Nonetheless, given the large number of ways where multimorbidity can be defined and measured, standards are needed if these are to be incorporated into trial reporting.

An interesting contrast between our study and most previous reports was our focus; we were concerned with all subgroup reports for trials regardless of whether they were reported in high impact journals. Underlying this difference is a difference in the consumer of the subgroups—the person looking at a single trial, versus the secondary researcher. For the reader of a single trial, to avoid dangers of overinterpretation, individual papers should be very cautious in reporting subgroup effects. However, this is the opposite of what is desirable for meta-analyses across multiple trials, where *completeness* and *consistency* would be helpful.

At present, neither audience is well served. As we show, trials are highly variable in what subgroups are reported, while as others have shown papers rarely meet the published standards for prespecification.^21 24 25^ In the digital age, both audiences could be served. Trial reports could limit subgroup reporting in line with current recommendations, while providing a wider common set of subgroup effect estimates via digital repositories in machine-readable formats using standard terminologies for secondary researchers. This is an exactly opposite strategy to reduce bias in subgroup reporting from that normally advocated—confining subgroups reporting to a small set of prespecified variables—instead rather we reduce bias through completeness. This would of course require an agreement as to what should constitute such a wider common set of subgroup effects (eg, consistent definition of subgroups, identification of important subgroups across different diseases, establishment of cut-off values for continuous subgroups especially for age, or model continuous variables as continuous variables and account for non-linearity by fractional polynomials or cubic splines). We hope that our findings, showing dramatic and unhelpful variation across trials, and a paucity of information on the impact of health states important for decision-making (such as comorbidities and frailty), help demonstrate a need for such a consensus.

Another way to improve subgroup analysis is through IPD meta-analysis (IPD-MA), considered as the gold standard for exploring subgroup effects.^26^ Patients with specific or combinations of characteristics can be identified through IPD across different studies, then combined in an MA. It offers increased power compared with individual studies,^27^ allows for better flexibility to standardise subgroup definitions and provides a higher credibility for findings compared with traditional MA.^26 28^ However, it suffers from some disadvantages such as requiring substantial resources to obtain IPD, clean and create consistent data format across studies, data quality issues, and it has not been widely adopted.^27 29^ Moreover, there are legal and ethical considerations regarding privacy and confidentiality when sharing IPD.^30^ Thus there are also challenges in accessing and using IPD to examine subgroup effects. Additionally, some frequently used regression-based methods in IPD-MA suffer from false positives.^26^ There is a trade-off between facilitating consistent subgroup reporting that would allow better meta-analysis of subgroups versus the increase in subgroup reporting which, if interpreted at the individual trial level, may lead to more false positives. Explicit guidance, reporting frameworks for subgroups should be developed to prevent misinterpretation and ensure the reliability of subgroup findings.

### Conclusion

Among 23% of trials reporting subgroups, age, sex, race/ethnicity and features of the index condition were the most common subgroups. Where subgroup effects for other conditions were reported, these were largely confined to the same body system as the index condition. Outside these areas, information on variation in treatment effects was sparse.

## supplementary material

10.1136/bmjopen-2023-081315 online supplemental file 1

10.1136/bmjopen-2023-081315 online supplemental file 2

10.1136/bmjopen-2023-081315 online supplemental file 3

## Data Availability

All data relevant to the study are included in the article or uploaded as supplementary information.
