# Supplementary material for: Description of subgroup reporting in clinical trials of chronic diseases: a meta-epidemiological study
Source: BMJ Open. 2024 Jun 20;14(6):e081315. doi: 10.1136/bmjopen-2023-081315 (PMC11328666; doi:10.1136/bmjopen-2023-081315)
Supplement: online supplemental file 1 [file bmjopen-14-6-s001.pdf]

A description of subgroup reporting in clinical trials of chronic diseases: a meta-epidemiological study.

Supplementary material

Lili Wei[1*], Elaine W Butterly[1], Jesús Rodríguez Pérez[1], Avirup Chowdhury[2], Richard Shemilt[1], Peter Hanlon[1], David A McAllister[1]

[1] School of Health and Wellbeing, University of Glasgow, UK

[2] The Institute of Cancer Research, UK

* Corresponding author

Dr Lili Wei

School of Health and Wellbeing, University of Glasgow

Clarice Pears Building, 90 Byres Road, G12 8TB, Glasgow, United Kingdom

Lili.Wei@glasgow.ac.uk

+44 (0) 141-330- 3299

Contents

Identifying trials, papers and subgroups. 3

Identifying eligible trials from clinicaltrials.gov. 3

Supplementary Table 1. Search criteria to identify trials from clinicaltrials.gov. 3

Supplementary Table 2. Included conditions, Medical Subject Heading (MeSH) terms and MeSH codes. 4

Screening eligible trials for reporting results. 7

Supplementary Figure 1. The screening of eligible trials with reported results. 7

Screening eligible trials/papers with reported results for reporting subgroups. 7

Obtaining standard format for tables obtained from eligible papers. 7

Assigning MeSH terms 8

Results. 8

Subgroup reporting summary statistics. 8

Supplementary Table 3. The proportion of subgroup reporting and commonest subgroups in each index condition. 8

Coefficients from regression models. 11

Supplementary Table 4.1. Coefficients from the total number of subgroups model. 11

Supplementary Table 4.2. Coefficients from subgroup reporting (any vs none) model. 12

Supplementary Table 4.3. Coefficients from overall results reporting (any vs none) model. 13

Identifying trials, papers and subgroups.

Identifying eligible trials from clinicaltrials.gov.

Supplementary Table 1. Search criteria to identify trials from clinicaltrials.gov.

Criteria	Trials
Start date >= 1990-01-01	16957
Study type – interventional.<br>Status – 'Active, not recruiting', 'Completed', 'Terminated'.<br>Phase – 2/3, 3 or 4<br>Enrolment >= 300 (or start date < '2005-01-01')<br>Participants not excluded for being 60 or older	13079
Study design is "Factorial Assignment", "Parallel Assignment" and "allocation random"	10841
Selected MeSH conditions *(see below)* found in study title or in browse conditions or conditions table	4348
Intervention type is "Biological" or "Drug"	3872

Conditions were eligible if the string (or the reverse of the string e.g. "Angina, Unstable" or "Unstable angina") corresponding to one or more the following MESH terms (or to a more specific terms in the MESH hierarchy) was found:
C05.116.198.579, C05.116.900.853.625.800, C05.550.114, C05.799.114, C05.799.414, C05.799.613, C06.405.117.119.500.204, C06.405.117.119.500.432, 06.405.117.119.500.450, C06.405.117.119.500.484, C06.405.117.119.500.484.500, C06.405.117.119.500.742, C06.405.117.620, C06.405.205.265.231, C06.405.205.731, C06.405.469.432, C06.405.608.348, C06.405.748.240, C06.405.748.398, C06.552.380.350.050, C08.127.108, C08.127.384, C08.127.446.567, C08.381.423, C08.381.483.487, C08.381.483.487.500, C08.381.495, C08.381.746, C08.381.765, C08.460.799, C08.674.095, C08.730.099.567, C09.603.799.315, C10.114.375.500, C10.228.140.079.862, C10.228.140.300.150, C10.228.140.300.275.800, C10.228.140.300.400, C10.228.140.300.510.200.325, C10.228.140.300.510.200.387, C10.228.140.300.510.200.418, C10.228.140.300.510.800.500, C10.228.140.300.775, C10.228.140.380.100, C10.228.140.380.230, C10.228.140.490, C10.228.140.546.399.750, C10.228.662.600, C10.574.812, C10.574.945.249, C10.803, C12.294.565.500, C12.777.419.192, C12.777.829.866, C12.777.934.284, C12.777.934.852, C13.351.968.829.813, C13.351.968.934.252, C13.351.968.934.814, C14.280.067.198, C14.280.067.248, C14.280.434, C14.280.647, C14.907.137.126.307, C14.907.137.126.307.500, C14.907.137.126.339, C14.907.137.126.372.500, C14.907.137.126.669, C14.907.253.092.477.200, C14.907.253.560.350.500, C14.907.253.855, C14.907.355, C14.907.355.350.700, C14.907.355.590, C14.907.355.830, C14.907.489, C14.907.585, C14.907.617, C17.300.480, C17.300.540, C17.300.775, C17.300.799, C17.800.784, C17.800.784.602, C17.800.784.801, C17.800.784.801.500, C17.800.859.675, C17.800.862.945, C18.452.394.750, C18.452.584.500.500.396, C18.452.584.500.500.438, C18.452.584.500.500.851, C18.452.648.398.450, C19.246.267, C19.246.300, C20.111.193, C20.111.197, C20.111.198, C20.111.199, C20.111.258.250.500, C20.111.327, C20.111.567, C20.543.480.680.095, C20.543.480.680.443, F03.087.400, or F03.675.700.
Using the normalised names, we used a combination of string comparison functions and manual review to restrict the set of trials to those where one or more arm-comparison compared eligible drugs (or classes), or compared an eligible drug to either placebo, usual-care or a "standard comparator".

After removing trials without eligible drugs and selecting enrolment >=300 (in free text fields), we got a final "denominator" dataset of 2235 clinical trials.

Supplementary Table 2. Included conditions, Medical Subject Heading (MeSH) terms and MeSH codes.

Category	MeSH term	Code
Musculoskeletal diseases [C05]	Osteoporosis	C05.116.198.579
	Spondyloarthropathies	C05.116.900.853.625 .800
	Arthritis	C05.550.114
	Arthritis, Rheumatoid Gout	C05.799.114
	Osteoporosis	C05.799.414
Digestive system diseases [C06]	CREST Syndrome	C06.405.117.119.500 .204
	Oesophageal Achalasia	C06.405.117.119.500 .432
	Oesophageal spasm, diffuse	C06.405.117.119.500 .450
	Gastro-oesophageal reflux	C06.405.117.119.500 .484
	Laryngopharyngeal reflux	C06.405.117.119.500 .484.500
	Plummer-Vinson Syndrome	C06.405.117.119.500 .742
	Oesophagitis	C06.405.117.620
	Colitis, Ulcerative	C06.405.205.265.231
	Inflammatory Bowel Diseases	C06.405.205.731
	Inflammatory Bowel diseases	C06.405.469.432
	Oesophagitis, peptic	C06.405.608.348
	Duodenogastric reflux	C06.405.748.240
	Gastritis	C06.405.748.398
	Hepatitis, autoimmune	C06.552.380.350.050
Respiratory Tract Diseases [C08]	Asthma	C08.127.108
	Bronchiectasis	C08.127.384
	Bronchitis, chronic	C08.127.446.567
	Hypertension, Pulmonary	C08.381.423
	Idiopathic Interstitial Pneumonias	C08.381.483.487
	Idiopathic Pulmonary Fibrosis	C08.381.483.487.500
	Lung Diseases, Obstructive	C08.381.495
	Pulmonary Embolism	C08.381.746
	Pulmonary Fibrosis	C08.381.765
	Rhinitis	C08.460.799
	Asthma	C08.674.095
	Bronchitis, Chronic	C08.730.099.567
Otorhinolaryngologic Diseases [C09]	Rhinitis, Allergic	C09.603.799.315

Nervous System Diseases [C10]	Multiple Sclerosis	C10.114.375.500
	Parkinsonian Disorders	C10.228.140.079.862
	Brain Ischaemia	C10.228.140.300.150
	Stroke, Lacunar	C10.228.140.300.275.800
	Dementia, Vascular	C10.228.140.300.400
	Infarction, Anterior Cerebral Artery	C10.228.140.300.510.200.325
	Infarction, Middle Cerebral Artery	C10.228.140.300.510.200.387
	Infarction, Posterior Cerebral Artery	C10.228.140.300.510.200.418
	Dementia, Vascular	C10.228.140.300.510.800.500
	Stroke	C10.228.140.300.775
	Alzheimer Disease	C10.228.140.380.100
	Dementia, Vascular	C10.228.140.380.230
	Epilepsy	C10.228.140.490
	Migraine Disorders	C10.228.140.546.399.750
	Parkinsonian Disorders	C10.228.662.600
	Parkinson Disease	C10.574.812
	Alzheimer Disease	C10.574.945.249
	Restless Leg Syndrome	C10.803
Urogenital Diseases [C12]	Prostatic Hyperplasia	C12.294.565.500
	Diabetic Nephropathies	C12.777.419.192
	Urinary Bladder, Overactive	C12.777.829.866
	Enuresis	C12.777.934.284
	Urinary Incontinence	C12.777.934.852
	Urinary Bladder, Overactive	C13.351.968.829.813
	Enuresis	C13.351.968.934.252
	Urinary Incontinence	C13.351.968.934.814
Cardiovascular Diseases [C14]	Atrial Fibrillation	C14.280.067.198
	Atrial Flutter	C14.280.067.248
	Heart Failure	C14.280.434
	Myocardial Ischaemia	C14.280.647
	Atherosclerosis	C14.907.137.126.307
	Peripheral Arterial Disease	C14.907.137.126.307.500
	Coronary Artery Disease	C14.907.137.126.339
	Dementia, Vascular	C14.907.137.126.372.500
	Intermittent Claudication	C14.907.137.126.669
	Cerebral Infarction	C14.907.253.092.477.200
	Dementia, Vascular	C14.907.253.560.350.500

	Stroke	C14.907.253.855
	Embolism and Thrombosis	C14.907.355
	Pulmonary Embolism	C14.907.355.350.700
	Thromboembolism	C14.907.355.590
	Thrombosis	C14.907.355.830
	Hypertension	C14.907.489
	Myocardial Ischaemia	C14.907.585
	Peripheral Vascular Diseases	C14.907.617
Skin and Connective Tissue Diseases [C17]	Lupus Erythematosus, Systemic	C17.300.480
	Mixed Connective Tissue Disease	C17.300.540
	Rheumatic Diseases	C17.300.775
	Scleroderma, Systemic	C17.300.799
	Scleroderma, Systemic	C17.800.784
	Scleroderma, Diffuse	C17.800.784.602
	Scleroderma, Limited	C17.800.784.801
	CREST Syndrome	C17.800.784.801.500
	Psoriasis	C17.800.859.675
	Urticaria	C17.800.862.945
Nutritional and Metabolic Diseases [C18]	Diabetes Mellitus	C18.452.394.750
	Hypercholesterolemia	C18.452.584.500.500 .396
	Hyperlipidaemia, Familial Combined	C18.452.584.500.500 .438
	Hypertriglyceridemia	C18.452.584.500.500 .851
	Hyperlipidaemia, Familial Combined	C18.452.648.398.450
Endocrine System Diseases [C19]	Diabetes Mellitus, Type 1	C19.246.267
	Diabetes Mellitus, Type 2	C19.246.300
Immune System Diseases [C20]	Anti-Neutrophil Cytoplasmic Antibody-Associated Vasculitis	C20.111.193
	Antiphospholipid Syndrome	C20.111.197
	Arthritis, Juvenile	C20.111.198
	Arthritis, Rheumatoid	C20.111.199
	Multiple Sclerosis	C20.111.258.250.500
	Diabetes Mellitus, Type 1	C20.111.327
	Hepatitis, Autoimmune	C20.111.567
	Asthma	C20.543.480.680.095
	Rhinitis, Allergic	C20.543.480.680.443

Screening eligible trials for reporting results.

Supplementary Figure 1. The screening of eligible trials with reported results.

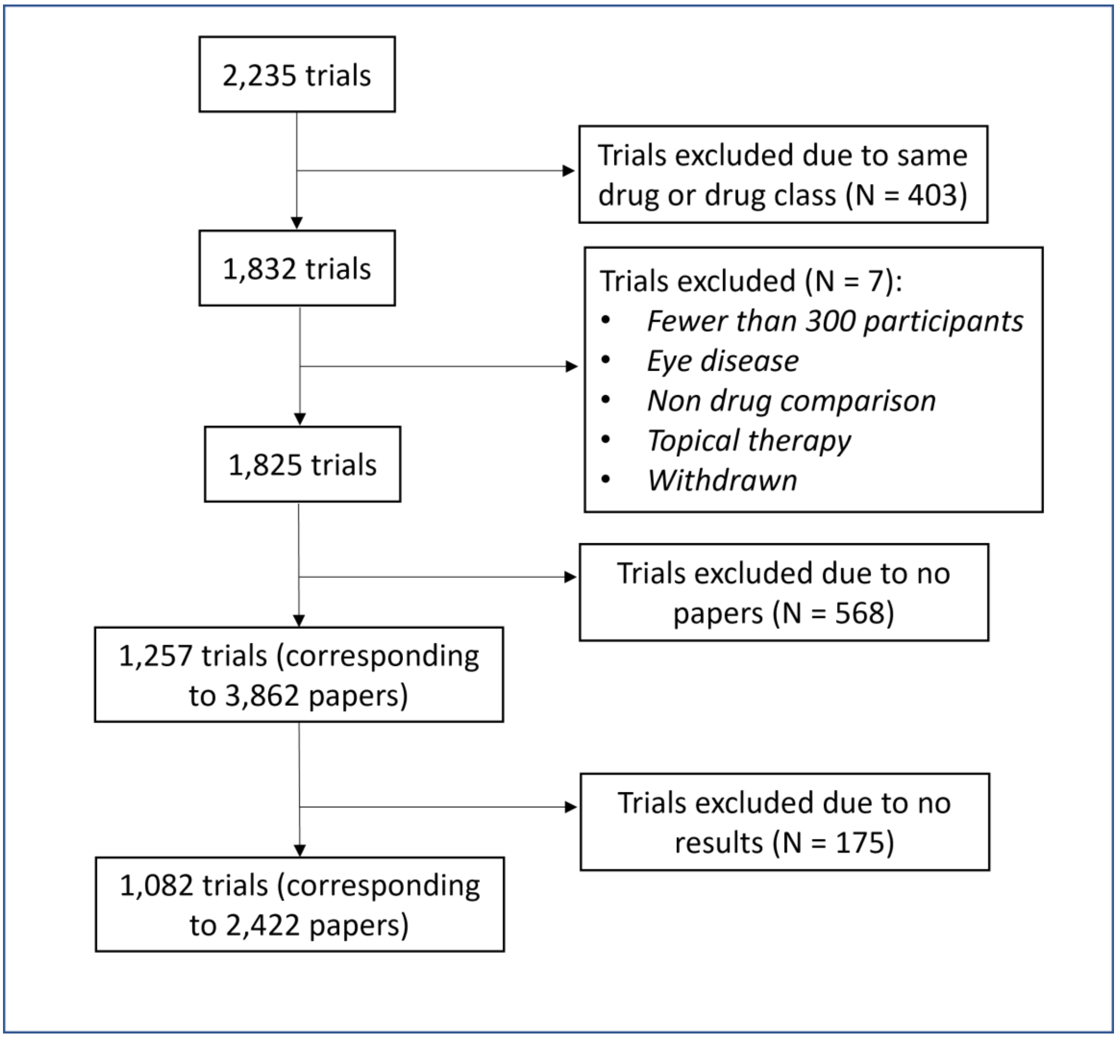

Screening eligible trials/papers with reported results for reporting subgroups.

2,422 papers with reported results obtained from the above screening process were then underwent the screening of subgroups analyses showed in Figure 1 in the main paper.

Obtaining standard format for tables obtained from eligible papers.

907 papers contain subgroup reporting after screening, as showed in Figure 1 in the main paper. Tables from these 907 papers in tabular format were uploaded to TableTidier (https://tabletidier.org/), a software designed to assist with extracting and standardising tables into a machine-readable format. Subsequently, each subgroup term was assigned a Concept Unique Identifier for a Metathesaurus concept (CUI). For example, if a table contains “sex” as a subgroup name and “woman” and “man” as subgroup levels, they are assigned CUI C0079399 [Gender Identity] as one subgroup. Therefore, synonyms used across the papers are harmonised allowing comparisons across different papers, trials and disease conditions.

Assigning MeSH terms

After obtaining CUIs for each subgroup, we harmonised them and assigned the closest MeSH or WHOATC code. Additionally, we supplemented certain CUIs with additional information if they included disease severity, duration, etc. Finally, these MeSH terms underwent clinical review.

Results.

Subgroup reporting summary statistics.

Supplementary Table 3. The proportion of subgroup reporting and commonest subgroups in each index condition.

Conditions	Total subgroups	The proportion of subgroup reporting among 2,235 trials n_T/N (%)	The proportion of subgroup reporting among 1,082 trials with results reporting n_R/N_R (%)	Five commonest subgroups in each condition
Myocardial Infarction	99	26/47 (55%)	25/30 (83%)	Age Factors (96.2%); Diabetes Mellitus (88.5%); Gender Identity (88.5%); **Myocardial Infarction (69.2%)**; Hypertension (30.8%)
Diabetes Mellitus, Type 2	89	120/460 (26%)	117/235 (50%)	Age Factors (49.17%); Glycated Hemoglobin A (48.33%); Gender Identity (39.17%); Body Mass Index (36.67%); Racial Groups (36.67%)
Coronary Artery Disease	77	27/80 (34%)	27/46 (59%)	Diabetes Mellitus (85.2%); Age Factors (74.1%); Gender Identity (74.1%); Myocardial Infarction (37.0%); Hypertension (33.3%)
Hypertension	64	44/247 (18%)	44/98 (45%)	Age Factors (59.1%); Gender Identity (52.3%); Diabetes Mellitus (38.6%); Racial Groups (36.4%); Blood Pressure (27.3%)
Heart Failure	51	17/40 (42%)	17/27 (63%)	Age Factors (70.6%); Diabetes Mellitus (64.7%); Gender Identity (64.7%); Stroke Volume (58.8%); **Heart Failure (52.9%)**
Hypercholesterolemia	48	28/72 (39%)	28/43 (65%)	Lipoproteins (71.4%); Diabetes Mellitus (67.9%); Age Factors (64.3%); Gender Identity (60.7%); Body Mass Index (53.6%)
Atrial Fibrillation	46	13/39 (33%)	13/20 (65%)	Age Factors (61.5%); Gender Identity (53.8%); Heart Failure (53.8%); **Atrial Fibrillation (46.2%)**; Hypertension (38.5%)
Pulmonary Disease, Chronic Obstructive	40	40/186 (22%)	39/96 (41%)	**Pulmonary Disease, Chronic Obstructive (75.0%)**; Age Factors (50.0%); Cigarette Smoking (45.0%); Gender Identity (42.5%); Steroids (40.0%)
Acute Coronary Syndrome	37	9/22 (41%)	9/10 (90%)	Age Factors (89%); Gender Identity (78%); Diabetes Mellitus (67%); Myocardial Infarction (56%); Percutaneous Coronary Intervention (56%)
Arthritis, Rheumatoid	35	28/106 (26%)	28/65 (43%)	**Arthritis, Rheumatoid (46.4%)**; Age Factors (25.0%); Gender Identity (21.4%);

				Immunosuppressive Agents (21.4%); C-Reactive Protein (17.9%)
Stroke	35	8/20 (40%)	8/13 (62%)	**Stroke (88%)**; Age Factors (62%); Gender Identity (62%); Diabetes Mellitus (38%); Hypertension (38%)
Atherosclerosis	30	2/9 (22%)	2/3 (67%)	Age Factors (100%); Body Mass Index (100%); Cigarette Smoking (100%); Diabetes Mellitus (100%); Gender Identity (100%)
Crohn Disease	29	11/18 (61%)	11/16 (69%)	Immunosuppressive Agents (63.6%); Tumor Necrosis Factor Inhibitors (63.6%); C-Reactive Protein (54.5%); **Crohn Disease (45.5%)**; Steroids (45.5%)
Osteoporosis	29	11/44 (25%)	11/23 (48%)	Age Factors (54.5%); Fractures, Bone (54.5%); **Osteoporosis (45.5%)**; Body Mass Index (27.3%); Geographic Locations (27.3%)
Prostatic Hyperplasia	28	9/30 (30%)	9/15 (60%)	Body Mass Index (44%); Age Factors (33%); Erectile Dysfunction (33%); Adrenergic alpha-Antagonists (22%); Antihypertensive Agents (22%)
Peripheral Arterial Disease	24	3/8 (38%)	3/4 (75%)	Diabetes Mellitus (67%); Age Factors (33%); Ankle Brachial Index (33%); Blood Pressure (33%); Body Weight (33%)
Venous Thromboembolism	23	7/36 (19%)	7/8 (88%)	Age Factors (86%); Gender Identity (86%); **Venous Thromboembolism (57%)**; Anticoagulants (43%); Body Weight (43%)
Asthma	22	19/147 (13%)	19/62 (31%)	**Asthma (31.6%)**; Eosinophilia (31.6%); Steroids (26.3%); Age Factors (21.1%); Gender Identity (21.1%)
Colitis, Ulcerative	21	8/14 (57%)	8/12 (67%)	Steroids (62%); Tumor Necrosis Factor Inhibitors (62%); C-Reactive Protein (38%); Gender Identity (38%); Age Factors (25%)
Psoriasis	19	13/62 (21%)	13/37 (35%)	Immunosuppressive Agents (38.5%); **Psoriasis (38.5%)**; Tumor Necrosis Factor Inhibitors (30.8%); Biological Therapy (15.4%); Cyclosporins (15.4%)
Diabetes Mellitus (unspecified)	16	8/36 (22%)	8/15 (53%)	Age Factors (75%); Body Mass Index (75%); Gender Identity (75%); Racial Groups (62%); Glycated Hemoglobin A (38%)
Osteoarthritis	14	6/64 (9%)	6/26 (23%)	Age Factors (50%); Arthritis, Rheumatoid (50%); Diabetes Mellitus (33%); Gender Identity (33%); Pain (33%)
Urticaria	12	2/3 (67%)	2/3 (67%)	Age Factors (50%); Angioedema (50%); Autoantibodies (50%); Body Weight (50%); Gender Identity (50%)
Diabetes Mellitus, Type 1	11	7/35 (20%)	7/17 (41%)	Glycated Hemoglobin A (57%); Insulin (43%); Age Factors (29%); Body Mass Index (29%); Glucose (29%)
Hyperlipidemias	11	1/7 (14%)	1/4 (25%)	Age Factors (100%); C-Reactive Protein (100%); Diabetes Mellitus (100%); Gender Identity (100%); Geographic Locations (100%)
Pulmonary Embolism	11	1/2 (50%)	1/1 (100%)	Age Factors (100%); Body Mass Index (100%); Fibrin Fibrinogen Degradation Products (100%); Gender Identity (100%); Neoplasms (100%)
Lupus Erythematosus, Systemic	10	4/8 (50%)	4/5 (80%)	Autoantibodies (50%); Racial Groups (50%); Steroids (50%); Albuminuria (25%); Antimalarials (25%)
Arthritis, Psoriatic	9	3/5 (60%)	3/4 (75%)	Immunosuppressive Agents (67%); Antirheumatic Agents (33%); Arthritis, Juvenile (33%); **Arthritis, Psoriatic (33%)**; Arthritis, Rheumatoid (33%)

Gastroesophageal Reflux	9	5/29 (17%)	5/8 (62%)	Body Mass Index (40%); Age Factors (20%); Gastrointestinal Diseases (20%); Gender Identity (20%); Heartburn (20%)
Seizures	9	6/31 (19%)	6/12 (50%)	Anticonvulsants (83%); Age Factors (50%); other antiepileptics (50%); Racial Groups (33%); Gender Identity (17%)
Spondylitis, Ankylosing	9	3/15 (20%)	3/8 (38%)	C-Reactive Protein (67%); Tumor Necrosis Factor Inhibitors (67%); Arthritis (33%); Cigarette Smoking (33%); Gender Identity (33%)
Angina Pectoris	8	2/4 (50%)	2/4 (50%)	Age Factors (100%); Gender Identity (100%); Body Weight (50%); Diabetes Mellitus (50%); Myocardial Infarction (50%)
Gout	8	5/11 (45%)	3/4 (75%)	Glomerular Filtration Rate (60%); Renal Insufficiency (60%); Age Factors (40%); Comorbidity (40%); Diuretics (40%)
Parkinson Disease	8	4/38 (11%)	4/12 (33%)	**Parkinson Disease (100%)**; Age Factors (75%); Gender Identity (75%); Body Weight (25%); Depression (25%)
Idiopathic Interstitial Pneumonias	7	3/8 (38%)	3/8 (38%)	Vital Capacity (67%); Age Factors (33%); Cigarette Smoking (33%); Geographic Locations (33%); Hydroxymethylglutaryl-CoA Reductase Inhibitors (33%)
Thromboembolism	7	1/4 (25%)	1/1 (100%)	Age Factors (100%); Embolism and Thrombosis (100%); Gender Identity (100%); Obesity (100%); Specialties, Surgical (100%)
Alzheimer Disease	6	4/31 (13%)	4/16 (25%)	**Alzheimer Disease (50%)**; Dementia (50%); Apolipoprotein A-I (25%); Gender Identity (25%); Mental Status and Dementia Tests (25%)
Multiple Sclerosis	6	2/8 (25%)	2/6 (33%)	Age Factors (100%); complex (100%); Coronary Artery Disease (50%); Gender Identity (50%); **Multiple Sclerosis (50%)**
Prediabetic State	6	1/1 (100%)	1/1 (100%)	Body Mass Index (100%); Body Weight (100%); Diabetes Mellitus (100%); Gender Identity (100%); Racial Groups (100%)
Venous Thrombosis	6	2/21 (10%)	2/5 (40%)	Age Factors (50%); Body Weight (50%); Gender Identity (50%); Neoplasms (50%); Renal Insufficiency (50%)
Ischemic Attack, Transient	5	1/1 (100%)	1/1 (100%)	Age Factors (100%); Coronary Artery Disease (100%); Gender Identity (100%); Racial Groups (100%)
Lupus Nephritis	5	1/4 (25%)	1/1 (100%)	Cyclophosphamide (100%); Gender Identity (100%); Geographic Locations (100%); Racial Groups (100%)
Spondylarthropathies	5	1/1 (100%)	1/1 (100%)	Age Factors (100%); Antirheumatic Agents (100%); Axial Spondyloarthritis (100%); Gender Identity (100%); Tumor Necrosis Factor Inhibitors (100%)
Migraine Disorders	3	2/22 (9%)	2/11 (18%)	Adrenergic beta-Antagonists (50%); **Migraine Disorders (50%)**; sumatriptan (50%)
Raynaud Disease	3	1/1 (100%)	1/1 (100%)	Blood Pressure (100%); Gender Identity (100%)
Retinal Vein Occlusion	2	1/4 (25%)	1/2 (50%)	Macular Edema (100%)
Rhinitis	2	2/41 (5%)	2/11 (18%)	Geographic Locations (50%)
Esophagitis	1	1/10 (10%)	1/1 (100%)	unclassifiable (100%)
Urinary Bladder, Overactive	1	1/39 (3%)	1/14 (7%)	Urinary Bladder Diseases (100%)

Some trials might correspond to multiple index conditions, we kept the commonest condition among 2,235 trials for simplicity; the number for some subgroups is the same in the 5th place and only one was kept based on the alphabetical order; the subgroup in bold is the subgroup same as the condition term with additional information such as type, severity, duration etc; $n_{T:}$ number of trials with subgroup reporting among 2,235 trials; $n_{R:}$ number of trials with subgroup reporting among 1,082 trials with results reporting; $N_{R:}$ trials with results reporting and N_R = 1,082.

Coefficients from regression models.

Supplementary Table 4.1. Coefficients from the total number of subgroups model.

Term	OR 95%CI
Start year	1.02 (1.02, 1.02)
Number of arms > 2	1.01 (0.99, 1.04)
log (enrolment, base = 10)	1.69 (1.65, 1.73)
Industry1	1 (0.97, 1.03)
Duration of follow up	1.03 (1.02, 1.03)
Acute Coronary Syndrome	1.43 (1.25, 1.64)
Alzheimer Disease	0.72 (0.4, 1.19)
Angina Pectoris	1.43 (0.94, 2.1)
Arthritis, Psoriatic	0.92 (0.68, 1.23)
Arthritis, Rheumatoid	1.45 (1.26, 1.67)
Atherosclerosis	2.12 (1.83, 2.46)
Atrial Fibrillation	2.11 (1.86, 2.4)
Colitis, Ulcerative	1.64 (1.38, 1.95)
Coronary Artery Disease	2.31 (2.06, 2.6)
Crohn Disease	3.09 (2.72, 3.53)
Diabetes Mellitus	1.2 (0.94, 1.51)
Diabetes Mellitus, Type 1	0.97 (0.72, 1.27)
Diabetes Mellitus, Type 2	2.3 (2.05, 2.58)
Esophagitis	0.29 (0, 2.11)
Gastroesophageal Reflux	0.23 (0.04, 0.69)
Gout	0.99 (0.72, 1.31)
Heart Failure	2.09 (1.85, 2.37)
Hypercholesterolemia	2.63 (2.34, 2.96)
Hypertension	1.87 (1.66, 2.12)
Idiopathic Interstitial Pneumonias	0.89 (0.5, 1.46)
Ischemic Attack, Transient	0.83 (0.45, 1.38)
Lupus Erythematosus, Systemic	1.04 (0.74, 1.43)
Lupus Nephritis	1.36 (0.81, 2.13)
Migraine Disorders	0.45 (0.08, 1.34)
Multiple Sclerosis	0.97 (0.5, 1.68)
Myocardial Infarction	2.19 (1.96, 2.47)
Osteoarthritis	1.39 (1.11, 1.73)
Osteoporosis	1.28 (1.08, 1.51)

Parkinson Disease	1 (0.7, 1.4)
Peripheral Arterial Disease	1.65 (1.44, 1.88)
Prediabetic State	0.87 (0.7, 1.07)
Prostatic Hyperplasia	2.85 (2.45, 3.31)
Psoriasis	1.12 (0.95, 1.33)
Pulmonary Disease, Chronic Obstructive	1.45 (1.29, 1.64)
Pulmonary Embolism	2.58 (1.92, 3.4)
Retinal Vein Occlusion	0.48 (0.14, 1.19)
Rhinitis	0.31 (0.01, 1.47)
Seizures	1.01 (0.75, 1.34)
Spondylarthropathies	1.26 (0.65, 2.18)
Spondylitis, Ankylosing	1.27 (0.91, 1.73)
Stroke	1.93 (1.64, 2.27)
Thromboembolism	1.67 (1.04, 2.52)
Urinary Bladder, Overactive	0.27 (0, 1.99)
Urticaria	2.84 (2.15, 3.7)
Venous Thromboembolism	1.43 (1.23, 1.68)
Venous Thrombosis	0.81 (0.63, 1.03)

Supplementary Table 4.2. Coefficients from subgroup reporting (any vs none) model.

Term	OR 95%CI
Start year	1.07 (1.03, 1.11)
Duration of follow up	1.13 (1.04, 1.24)
Number of arms > 2	1 (0.73, 1.37)
log (enrolment, base = 10)	3.48 (2.25, 5.47)
Industry1	1.58 (0.94, 2.69)
Acute Coronary Syndrome	10.44 (1.57, 210.5)
Alzheimer Disease	1.12 (0.27, 4.05)
Angina Pectoris	5.22 (0.57, 48.21)
Arthritis, Psoriatic	7.62 (0.87, 163.78)
Arthritis, Rheumatoid	1.66 (0.75, 3.73)
Atherosclerosis	5.46 (0.3, 143.72)
Atrial Fibrillation	4.26 (1.37, 14.07)
Colitis, Ulcerative	5.12 (1.39, 21.75)
Coronary Artery Disease	3.44 (1.34, 9.09)
Crohn Disease	7.06 (1.92, 30.14)
Diabetes Mellitus	4.05 (1.14, 14.81)
Diabetes Mellitus, Type 1	1.6 (0.49, 5.1)
Diabetes Mellitus, Type 2	2.44 (1.31, 4.72)
Gastroesophageal Reflux	4.19 (0.81, 24.01)
Gout	8 (0.93, 170.12)
Heart Failure	3.06 (1.08, 9.01)
Hypercholesterolemia	4.96 (2.09, 12.26)
Hypertension	2.48 (1.21, 5.22)

Idiopathic Interstitial Pneumonias	1.7 (0.31, 7.94)
Lupus Erythematosus, Systemic	8.85 (1.18, 181.81)
Migraine Disorders	1.1 (0.15, 5.07)
Multiple Sclerosis	0.53 (0.03, 4.17)
Myocardial Infarction	9.86 (2.94, 40.48)
Osteoarthritis	0.85 (0.24, 2.63)
Osteoporosis	2.45 (0.82, 7.34)
Parkinson Disease	2.18 (0.49, 8.84)
Peripheral Arterial Disease	2.79 (0.15, 77.92)
Prostatic Hyperplasia	3.92 (1.19, 13.71)
Psoriasis	0.84 (0.31, 2.2)
Pulmonary Disease, Chronic Obstructive	1.24 (0.6, 2.61)
Retinal Vein Occlusion	3.48 (0.13, 95.06)
Rhinitis	0.91 (0.13, 4.18)
Seizures	3.85 (0.99, 15.59)
Spondylitis, Ankylosing	1.87 (0.34, 8.97)
Stroke	3.85 (0.77, 22.09)
Urinary Bladder, Overactive	0.15 (0.01, 0.88)
Urticaria	7.49 (0.67, 168.83)
Venous Thromboembolism	6.99 (0.86, 150.52)
Venous Thrombosis	1.87 (0.22, 13.33)

Supplementary Table 4.3. Coefficients from overall results reporting (any vs none) model.

Term	OR 95%CI
Start year	0.97 (0.95, 0.99)
Duration of follow up	1.1 (1.03, 1.18)
Number of arms > 2	1.42 (1.15, 1.74)
log (enrolment, base = 10)	1.63 (1.22, 2.19)
Industry1	1.03 (0.73, 1.45)
Acute Coronary Syndrome	0.93 (0.35, 2.43)
Alzheimer Disease	1.15 (0.51, 2.56)
Arthritis, Psoriatic	4.2 (0.58, 84.42)
Arthritis, Rheumatoid	1.71 (1, 2.94)
Atherosclerosis	0.54 (0.11, 2.24)
Atrial Fibrillation	1.03 (0.49, 2.21)
Brain Ischemia	0.61 (0.02, 16.09)
Cerebral Infarction	0.42 (0.09, 1.54)
Colitis, Ulcerative	7.26 (1.87, 48.04)
Coronary Artery Disease	1.2 (0.65, 2.23)
Crohn Disease	7.85 (2.04, 51.75)
Diabetes Mellitus	0.76 (0.34, 1.66)
Diabetes Mellitus, Type 1	1.37 (0.64, 2.93)
Diabetes Mellitus, Type 2	1.41 (0.95, 2.08)
Diabetic Nephropathies	0.4 (0.02, 3.2)

Enuresis	0.51 (0.02, 4.08)
Esophagitis	0.13 (0.01, 0.72)
Gastroesophageal Reflux	0.48 (0.18, 1.18)
Gout	0.63 (0.16, 2.21)
Heart Failure	1.96 (0.92, 4.32)
Hypercholesterolemia	1.7 (0.95, 3.07)
Hyperlipidemias	1.01 (0.13, 6.37)
Hypertension	0.94 (0.6, 1.45)
Lupus Erythematosus, Systemic	1.8 (0.42, 9.18)
Lupus Nephritis	0.31 (0.02, 2.6)
Migraine Disorders	1.87 (0.72, 4.98)
Multiple Sclerosis	2.46 (0.5, 17.89)
Myocardial Infarction	1.8 (0.85, 3.9)
Osteoarthritis	0.94 (0.49, 1.77)
Osteoporosis	1.22 (0.59, 2.52)
Parkinson Disease	0.62 (0.26, 1.4)
Peripheral Arterial Disease	0.75 (0.13, 4.48)
Prostatic Hyperplasia	1.56 (0.68, 3.59)
Psoriasis	1.68 (0.9, 3.17)
Pulmonary Disease, Chronic Obstructive	1.48 (0.93, 2.36)
Pulmonary Embolism	0.82 (0.03, 21.15)
Restless Legs Syndrome	1.55 (0.46, 5.23)
Retinal Vein Occlusion	1.31 (0.15, 11.48)
Rhinitis	0.48 (0.21, 1.03)
Seizures	0.68 (0.29, 1.56)
Spondylitis, Ankylosing	1.38 (0.46, 4.2)
Stroke	1.45 (0.43, 5.26)
Thromboembolism	0.52 (0.02, 5.61)
Urinary Bladder, Overactive	0.78 (0.35, 1.67)
Venous Thromboembolism	0.26 (0.08, 0.68)
Venous Thrombosis	0.31 (0.1, 0.87)
