# Supplementary figures and images for: Description of subgroup reporting in clinical trials of chronic diseases: a meta-epidemiological study
Source: BMJ Open. 2024 Jun 20;14(6):e081315. doi: 10.1136/bmjopen-2023-081315 (PMC11328666; doi:10.1136/bmjopen-2023-081315)

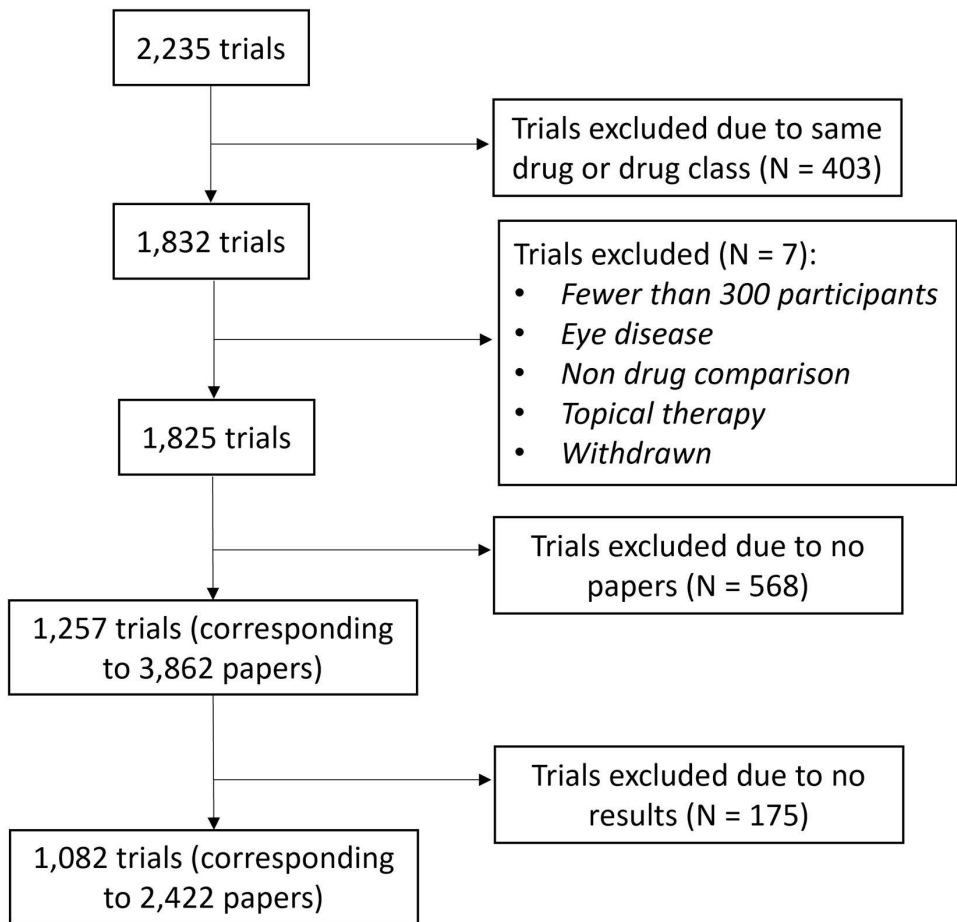

Supplement: online supplemental file 2 [file bmjopen-14-6-s002.pdf]
