# Supplementary material for: Description of subgroup reporting in clinical trials of chronic diseases: a meta-epidemiological study
Source: BMJ Open. 2024 Jun 20;14(6):e081315. doi: 10.1136/bmjopen-2023-081315 (PMC11328666; doi:10.1136/bmjopen-2023-081315)
Supplement: Supplementary file 4 [file bmjopen-2023-081315.reviewer_comments.pdf]

PEER REVIEW HISTORY

BMJ Open publishes all reviews undertaken for accepted manuscripts. Reviewers are asked to complete a checklist review form (**http://bmjopen.bmj.com/site/about/resources/checklist.pdf**) and are provided with free text boxes to elaborate on their assessment. These free text comments are reproduced below.

ARTICLE DETAILS

**TITLE (PROVISIONAL)**	A description of subgroup reporting in clinical trials of chronic diseases: a meta-epidemiological study.
**AUTHORS**	Wei, Lili; Butterly, Elaine; Rodríguez Pérez, Jesús; Chowdhury, Avirup; Shemilt, Richard; Hanlon, Peter; McAllister, David

VERSION 1 – REVIEW

**REVIEWER**	Schoenenberger, Christof Manuel<br>Universitatsspital Basel, Division of clinical Epidemiology
**REVIEW RETURNED**	01-Dec-2023

**GENERAL COMMENTS**	Thank you for the opportunity to comment on this interesting paper. I think the topic of subgroups is very important in methodological research and I find some of the approaches used by the authors very innovative. The study is overall well reported. However, I think the authors should try to report the basically intuitive results more clearly and not complicate them with too complex figures. Moreover, the topic of individual patient data meta-analysis (IPDMA) should also be addressed when it comes to better capturing subgroup effects in evidence syntheses. I have tried to list some points and hopefully contribute positively to the manuscript:<br><br>1) The paragraph in the introduction where you write about the development of a standard subgroup set is a bit missing context. There are other and more important factors to determine subgroups than the frequency of subgroups used so far.<br>2) It is not clear to me how you selected the trials for this project. Even after consulting the study protocol, these questions remain unanswered. I think it would be important to describe this in more detail, especially as the introduction suggests that this is a very comprehensive sample. A justification is also required for the clinical conditions included.<br>3) Did I understand correctly that information about the subgroups was extracted exclusively from results publications and not from the trial register or published protocols? This should be described more explicitly in the methods section.<br>4) The date of the last update search for publications is 4.5 years back. Is there a reason for this?<br>5) I don't know the tabletider tool. Is there some validation for it?<br>6) Very nice that the R code is available via GitHub.<br>7) Table1:

	a. Are these absolute numbers in the column “five commonest subgroups in each condition”? If yes, would percentages not make more sense to compare between different conditions?<br>8) Figure 2:<br>a. I have trouble interpreting Figure 2 as there is no information on how many trials are used for each analysis.<br>b. What is about the variable “start year”. What is the hypothesis here?<br>c. The categorization for medical conditions makes sometimes no sense to me, e.g. why should a lupus nephritis be musculoskeletal?<br>d. Overall, I think this figure should be simplified and less information should be presented in this one figure. It is too much for the reader.<br>9) Figure 3:<br>a. What is the reasoning behind showing the empty rows, i.e. number of trials equals zero.<br>b. I find it difficult to draw conclusions from this figure because these "disease systems" are very abstract. For example, what does C06/C17 worth 1.4 mean? What are the concrete trial/medical conditions here? I understand the basic intention behind this figure, but the fact that CVD trials more often use diabetes as a subgroup seems relatively intuitive to me. So, I'm not sure what added value this figure provides.<br>10) “With the exception of cardiometabolic and thromboembolic diseases, and especially for subgroups not closely related to the index condition - the published literature contains only sparse information on how treatment effects differ within trials.” I don’t understand what you mean with the last part. What published literature? The trials from your sample or existing methodological literature?<br>11) I would avoid the term chronic medical condition since there is no definition for it.<br>12) I would suggest adding following paper to the discussion. Taji Heravi A, Gryaznov D, Schandelmaier S, Kasenda B, Briel M, Adherence to SPIRIT Recommendations (ASPIRE) Study Group. Evaluation of Planned Subgroup Analysis in Protocols of Randomized Clinical Trials. JAMA Netw Open. 2021;4(10):e2131503. doi:10.1001/jamanetworkopen.2021.31503<br>13) I don’t understand why it is a main finding the frailty and mental health are rare subgroups. This seems to make sense due to the studied conditions.<br>14) Regarding the commonest subgroups reported: I'm not sure if it makes sense to list diabetes and HbA1c separately.<br>15) Personal preference, but I would try to keep the number of abbreviations as low as possible, for example write myocardial infarction instead of MI.<br>16) Table S2: Why are neurological diseases listed under Otorhinolaryngologic diseases? Also, some diseases as stroke are listed under cardiovascular as well as under Otorhinolaryngologic.<br>17) I can understand the suggestion made towards the end of the discussion to make "wider common set of subgroups accessible via digital repositories". However, the effort involved seems immense to me and not really feasible. In my opinion, the solution for better subgroup analyses is closer, namely in the form of IPDMAs. This aspect should be included in the discussion.

REVIEWER	Haller, Bernhard<br>Technische Universität München
REVIEW RETURNED	23-Jan-2024

| GENERAL COMMENTS | I reviewed the article „A description of subgroup reporting in clinical trials of medical conditions“ by Wei et al. In the article, an overview over performed subgroup analyses in clinical trials is given. While this is an interesting article I have some major points of critique.<br><br>Some of the aspects in the article are not described precisely enough, so it is hard to understand how the data were exactly extracted and analysed. Amongst others, this refers to the following aspects:<br>- The regression models that were fitted to the data should be described in more detail (in the methods and the results section). It was not clear to me, whether univariate or multivariable models were fitted to the data. Additionally, some important details as e.g. the unit and differences the estimated odds and rate ratios refer to are not given in the corresponding figure, but only in the text (“per 10-fold increase”, per year of follow-up”). Reference groups are only mentioned in the text, but are not shown in the figure or given in the abstract.<br>- Much more information should be presented for the heatmap(s) that can be generated on the linked websites. I was not able to understand what is produced on the website, what the resulting heatmaps show, and what the given filters really mean. Additionally, I was not able to use more than two filters.<br>- The outcomes that were used. What does “reporting of any results” mean. Additionally, what is one subgroup in your analysis? Is presentation of results for men and women one subgroup (“sex”) or two (“men”, “women”)?<br><br>I like the motivation and the discussion with the reference to meta-analysis and the dilemma between “over-interpretation” and “completeness”. I also like the suggestion of establishing common subgroups that should be reported and of providing results in digital repositories. But there are two aspects that I miss in the discussion (and also in the results). While it is mentioned that age was one of the most common variables for subgroup definition, the cut-off values used for definition of subgroups are not mentioned in the manuscript. In order to use age subgroups in a meta-analysis, the same (or at least similar) age subgroups had to be considered in the analysis of clinical trials. A second aspect that, in my opinion, should be discussed in more detail is the increased risk of false-positive findings due to multiplicity when more subgroups are investigated for the matter of completeness (which might include subgroups where not biological plausibility for treatment effect heterogeneity exists).<br><br>I have some additional minor comments:<br>- It is mentioned that this “was last updated in 04/2019”. Does this refer to the search for publications of the trials? That would have been quite a long time ago. - I think Figure 3 is a bit hard to read and interpret. The figure caption should provide more details or the figure should be adapted. The text refers to the “diagonals”, but as |

	the entries on the x-axis and on the y-axis are not identical, so there is no real diagonal.<br>- In the discussion, the following sentence can be found: “Alternatively, our null association for industry funding could be due to heterogeneity according to statistical significance of the primary outcome.” I do not really understand what is meant here. Could you please explain and/or rephrase the sentence?<br>- Labels in figures are generally too small.<br>- I believe the terms “sponsor” and “funder” are used interchangeably, but should be distinguished.

VERSION 1 – AUTHOR RESPONSE

Reviewer #1: Dr. Christof Manuel Schoenenberger, Universitatsspital Basel

Thank you for the opportunity to comment on this interesting paper. I think the topic of subgroups is very important in methodological research and I find some of the approaches used by the authors very innovative. The study is overall well reported. However, I think the authors should try to report the basically intuitive results more clearly and not complicate them with too complex figures. Moreover, the topic of individual patient data meta-analysis (IPDMA) should also be addressed when it comes to better capturing subgroup effects in evidence syntheses. I have tried to list some points and hopefully contribute positively to the manuscript.

Thank you for your interest and enthusiasm about our work, and insightful suggestions. We really appreciate your acknowledgement in the importance of subgroup work in methodological research. We have extensively revised the manuscript in response to these which we believe has led to an improved revised manuscript.

1) The paragraph in the introduction where you write about the development of a standard subgroup set is a bit missing context. There are other and more important factors to determine subgroups than the frequency of subgroups used so far.

Thank-you. We have amended the text to reflect the importance of other factors as follows:-

Page 4, para3:

“Alongside other important considerations such as clinical factors, biological plausibility and statistical constraints, this could also inform the development of a standard set subgroups for different index conditions and interventions”

2) It is not clear to me how you selected the trials for this project. Even after consulting the study

protocol, these questions remain unanswered. I think it would be important to describe this in more detail, especially as the introduction suggests that this is a very comprehensive sample. A justification is also required for the clinical conditions included.

Thanks for pointing this out. More details have been added.

Page 5, para 1:

The selection criteria include phase 2/3, 3, or 4 trials, recruiting ≥ 300 participants, with an upper age limit of ≥ 60 years or no maximum(2) (see Table S1 in the appendix). Conditions were chosen based on the requirement for long-term pharmacological therapy, including a range of cardiovascular…

3) Did I understand correctly that information about the subgroups was extracted exclusively from results publications and not from the trial register or published protocols? This should be described more explicitly in the methods section.

Yes. It has been added.

Page 5, para 4:

Subgroup data was extracted exclusively from publications. In our experience subgroup results are rarely added to clincialtrials.gov.

4) The date of the last update search for publications is 4.5 years back. Is there a reason for this?

Thank-you for this question. This work involved a number of highly time-consuming steps including extracting all subgroup data in text, figures and tables (performing Optical Character Recognition where needed), identifying over 2,000 unique strings with subgroup descriptions, mapping these across to UMLS and then MeSH terms and finally clinical review of all terms. The initial stages involved help from more than 15 individuals. As such, it would not be practical to update the search. However, we do not believe that this poses a serious limitation as the work is intended to provide a broad overview of subgroup reporting rather than contemporary evidence about a specific clinical therapeutic decision. Moreover, we are unaware of any recent change in subgroup reporting guidance that is likely to have caused a difference.

5) I don’t know the tabletider tool. Is there some validation for it?

Thanks for this question. TableTidier is a tool for cleaning and transforming tabular data. However, while it uses machine learning algorithms, these are simply to reduce point-and-click type interactions (eg such are typically used when reshaping data in excel) with human beings fully supervising the

process and hence (unlike automatic tools) validation is not required. We are hoping to undertake some work with TableTidier to determine whether or not it saves researcher's time, but this has yet to be completed. TableTidier is documented here - https://tabletidier.org/.

6) Very nice that the R code is available via GitHub.

Thank-you.

7) Table1:
a. Are these absolute numbers in the column "five commonest subgroups in each condition"? If yes, would percentages not make more sense to compare between different conditions?

Thank-you. These have been changed into percentages.

8) Figure 2:
a. I have trouble interpreting Figure 2 as there is no information on how many trials are used for each analysis.

Thank-you. This has been added.

Page 7, para 3:

Figure 2 shows associations for any overall result reporting (yes/no among 2,235 trials), any subgroup reporting (yes/no among 1,082 trials reporting overall results) using logistic regression models.

b. What is about the variable "start year". What is the hypothesis here?

b. This is the trial starting year; we wanted to find out if more recently conducted trials tend to have a better design and reporting for subgroup analysis. We found no change over the 28 years of the trial registrations from 1990 to 2017.

c. The categorization for medical conditions makes sometimes no sense to me, e.g. why should a lupus nephritis be musculoskeletal?

We apologise, lupus nephritis and lupus erythematosus have been moved into "Other diseases".

d. Overall, I think this figure should be simplified and less information should be presented in this one figure. It is too much for the reader.

Thanks for this comment, we agree that the figure is unclear and have simplified the figure in a number of ways :-

- removed the total number of subgroups from figure 2 to table S4.2 in the appendix (page 13)
- split Figure 2 into 2a) chronic diseases and 2b) trial characteristics

- Figure 2. The associations between a) chronic diseases, b) trial characteristics with subgroup reporting and overall results reporting.

2a) Chronic diseases

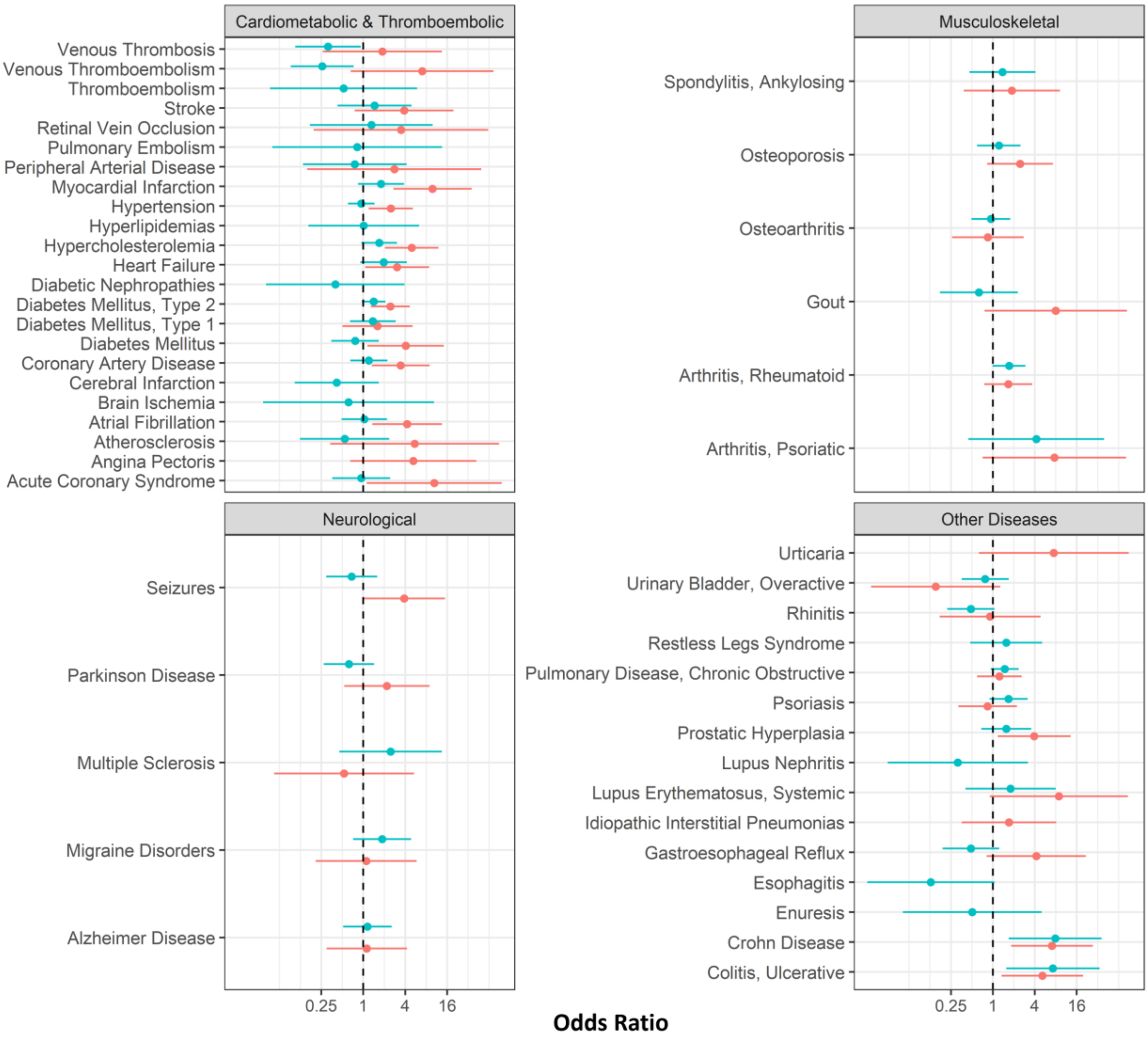

2b) Trial characteristics

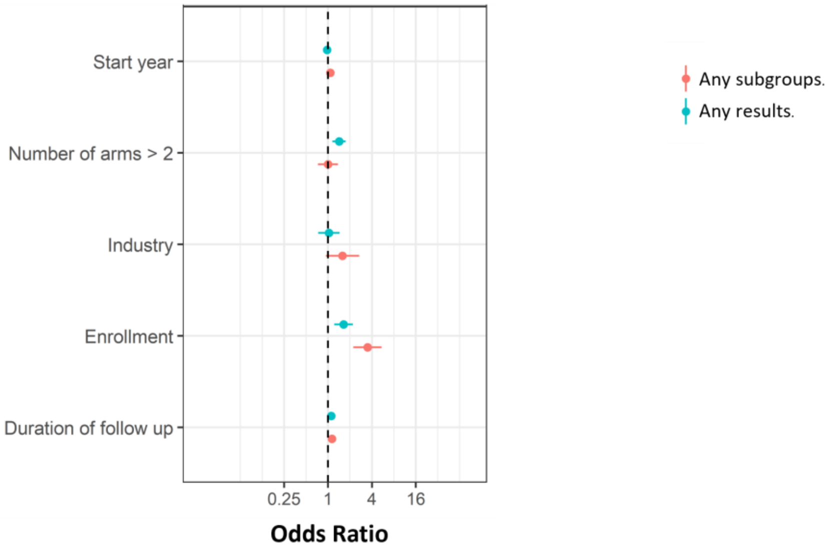

9) Figure 3:
a. What is the reasoning behind showing the empty rows, i.e. number of trials equals zero.

We apologise. The empty rows have been removed.

b. I find it difficult to draw conclusions from this figure because these "disease systems" are very abstract. For example, what does C06/C17 worth 1.4 mean? What are the concrete trial/medical conditions here? I understand the basic intention behind this figure, but the fact that CVD trials more often use diabetes as a subgroup seems relatively intuitive to me. So, I'm not sure what added value this figure provides.

We apologise, C06/C17 was a minor mistake and we have corrected it. Combining with another reviewer's comments, we added red borders for conditions and subgroups with the same organ system. The idea behind this plot is to show trials are more likely to report subgroups confined to conditions within the same body system. Apart from this, CVD trials are more likely to report diabetes, kidney disease and vice versa.

Figure 3. Comorbidity subgroup reporting.

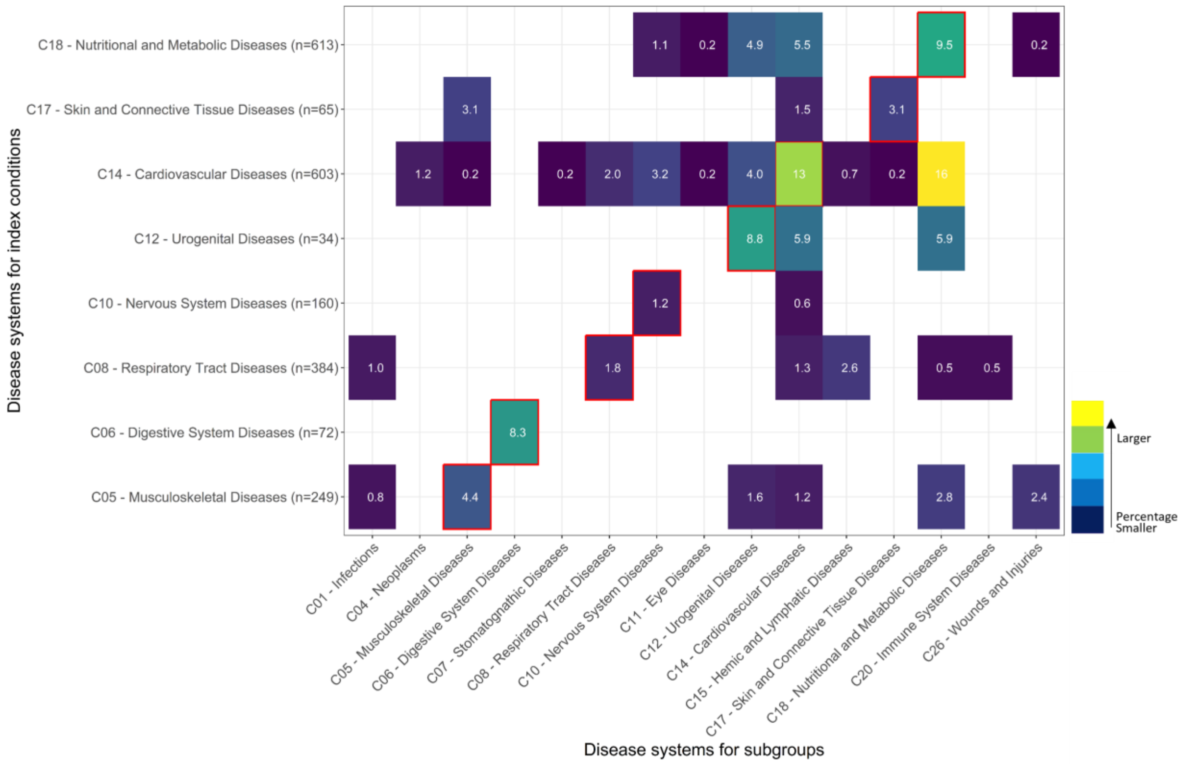

10) "With the exception of cardiometabolic and thromboembolic diseases, and especially for subgroups not closely related to the index condition - the published literature contains only sparse

information on how treatment effects differ within trials." I don't understand what you mean with the last part. What published literature? The trials from your sample or existing methodological literature?

We mean from our findings from corresponding published literature for those 2,235 trials. We have re-worded it into "With the exception of cardiometabolic and thromboembolic diseases, and especially for subgroups not closely related to the index condition - the published literature we screened contains only sparse information on how treatment effects differ within trials."

11) I would avoid the term chronic medical condition since there is no definition for it.

Thanks. We will use chronic diseases instead, which has a definition from WHO and more commonly used in the literature(3).

12) I would suggest adding following paper to the discussion. Taji Heravi A, Gryaznov D, Schandelmaier S, Kasenda B, Briel M, Adherence to SPIRIT Recommendations (ASPIRE) Study Group. Evaluation of Planned Subgroup Analysis in Protocols of Randomized Clinical Trials. JAMA Netw Open. 2021;4(10):e2131503. doi:10.1001/jamanetworkopen.2021.31503

Thanks. It has been added. Page 10, para 2:

Taji et al also showed that trial protocols with subgroup planning were more likely to be industry-sponsored than those without planned subgroup(4).

Page 10, para 3:

Even in examining trial protocols, age and sex are the most frequently planned subgroups(4).

13) I don't understand why it is a main finding the frailty and mental health are rare subgroups. This seems to make sense due to the studied conditions.

Thanks for this. We believe that the main findings should include not only commonly reported subgroups but also those that are less frequently reported. Considering that comorbidity, multimorbidity, and frailty are significant prognostic factors for mortality and/or reduced quality of life, mental health status plays an important role in chronic diseases. The scarcity of reporting on these subgroups is an important finding to inform future trial design and subgroup pre-specifications. There is increasing interest and importance in determining whether treatment strategies should be altered in the presence of frailty/comorbidity(5). Additionally, the adaptation and personalisation of mental health treatments to cater to different groups are gaining increasing attention to enhance their acceptability and benefits to patients(6). Subgroup reporting of these could be an important part of this evidence.

14) Regarding the commonest subgroups reported: I'm not sure if it makes sense to list diabetes and HbA1c separately.

We think we should respect the original paper to keep it separate. For example, this stroke trial(7) excluded patients with diabetes but included HbA1c as a subgroup for its primary outcome.

15) Personal preference, but I would try to keep the number of abbreviations as low as possible, for example write myocardial infarction instead of MI.

Thanks. Changes have been made in page 8, para 2.

16) Table S2: Why are neurological diseases listed under Otorhinolaryngologic diseases? Also, some diseases as stroke are listed under cardiovascular as well as under Otorhinolaryngologic.

We apologise for this error. The label for Nervous System Diseases [C10] which is supposed to be just under Otorhinolaryngologic Diseases [C09] was missed in the Table processing and it has now been added back in. Those neurological diseases should be under Nervous System Diseases [C10].

17) I can understand the suggestion made towards the end of the discussion to make "wider common set of subgroups accessible via digital repositories". However, the effort involved seems immense to me and not really feasible. In my opinion, the solution for better subgroup analyses is closer, namely in the form of IPDMAs. This aspect should be included in the discussion.

As researchers who use trial IPD in our own research, we are not sure that we agree that IPD presents an easier solution. Access to IPD requires, often complex, data sharing agreements, dealing with redaction (as part of anonymisation procedures) and huge amounts of time in data cleaning, analysis and even export from trusted research environments. We agree however that this is an alternative to standard subgroup reporting and have added this to the discussion.

Thank-you. We have added this discussion. Page 11, para 4:

“Another way to improve subgroup analysis is through individual participant data meta-analysis (IPD-MA), considered as the gold standard for exploring subgroup effects(8). Patients with specific or combinations of characteristics can be identified through IPD across different studies, then combined in a MA. It offers increased power compared to individual studies(9), allows for better flexibility to standardise subgroup definitions, and provides a higher credibility for findings compared to traditional MA(8, 10). However, it suffers from some disadvantages such as requiring substantial resources to obtain IPD, clean and create consistent data format across studies, data quality issues, and it has not been widely adopted (9, 11). Additionally, some frequently used regression-based methods in IPD-MA suffer from false positives(8). There is a trade-off between facilitating consistent subgroup reporting

that would allow better meta-analysis of subgroups versus the increase in subgroup reporting which, if interpreted at the individual trial level, may lead to more false positives. Explicit guidance, reporting frameworks for subgroups should be developed to prevent misinterpretation and ensure the reliability of subgroup findings."

Reviewer #2: Dr. Bernhard Haller, Technische Universität München

I reviewed the article „A description of subgroup reporting in clinical trials of medical conditions" by Wei et al. In the article, an overview over performed subgroup analyses in clinical trials is given. While this is an interesting article I have some major points of critique.

Some of the aspects in the article are not described precisely enough, so it is hard to understand how the data were exactly extracted and analysed. Amongst others, this refers to the following aspects:

1) The regression models that were fitted to the data should be described in more detail (in the methods and the results section). It was not clear to me, whether univariate or multivariable models were fitted to the data. Additionally, some important details as e.g. the unit and differences the estimated odds and rate ratios refer to are not given in the corresponding figure, but only in the text ("per 10-fold increase", per year of follow-up"). Reference groups are only mentioned in the text, but are not shown in the figure or given in the abstract.

Thanks for pointing out that. More details about the models have been added to page 6, para 3:

We fitted two sets of logistic regression models for i) any overall results reported (if trials reported trial results at all) and ii) any subgroups reported (taking those with any overall results reported as the denominator). For both outcomes, multivariate regression models were used. Variables included were the year the trial started, number of arms ((>2 arms vs ≤2 arms), number of participants enrolled (log-transformed with a base of 10 such that the coefficient corresponds is to a 10-fold increment in sample size), sponsor type (industry vs other), duration of follow-up and the index condition (Table 1). These variables were mutually adjusted. The coefficients were presented on the exponential scale. Among trials with any subgroup reporting, we used quasi-Poisson models to examine the total number of subgroups. 'One subgroup' indicates whether there are multiple levels (e.g., 'age' includes both <65 and >65-year-olds, 'sex' includes both women and men), we count each subgroup only once. For the quasi-Poisson model, outcome was the count of subgroups per trial, and covariates included were the same as regression models.

For the unit, for every 10-fold increase in enrolment sample size (e.g., from 10 to 100, or from 100 to 1,000), the odds of reporting subgroups will be 3.48 times higher. The unit for starting year and

duration of follow-up is per year. For every year increase in the duration of follow-up, the odds of reporting subgroups will be 1.13 times higher. We also added details for this. Page 7, para 3:

"…odds ratio (OR) and 95% CI per 10-fold increase in number enrolled e.g., from 10 to 100: 1.63, 1.22 - 2.19… Duration of follow-up also predicted any result reporting (OR per year increase in follow-up 1.10, 1.03 - 1.18)…"

For the condition, asthma is the reference, and it won't be displayed in the figure as it is 1. We have added more details in the abstract. Page 2, para 4:

"…specific index conditions (e.g., hypercholesterolemia, hypertension, taking asthma as the reference, OR ranged from 0.15 to 10.44)…"

2) Much more information should be presented for the heatmap(s) that can be generated on the linked websites. I was not able to understand what is produced on the website, what the resulting heatmaps show, and what the given filters really mean. Additionally, I was not able to use more than two filters.

Thanks. The kind of data manipulation that can be performed using the interactive heatmaps is more typically done using statistical software, therefore despite considerable efforts to make it as useable as possible we agree that it is complex. To make the tool easier to use we have recorded a video demonstration and uploaded to the appendix. Please note that the original data can also be downloaded and aggregated/plotted in whichever ways other researchers find useful.

---

3) The outcomes that were used. What does "reporting of any results" mean. Additionally, what is one subgroup in your analysis? Is presentation of results for men and women one subgroup ("sex") or two ("men", "women")?

Thank you for asking this clarification. Among 2,235 trials identified from the clinicaltrials.gov (Figure S1 in appendix), some were excluded due to no drug comparisons, no publications, no results reported etc., only 1,082 trials reported trial results at all (we call this reporting of any overall results). We have added a definition of "reporting any results" to the methods section. Page 6, para 3:

We fitted two sets of logistic regression models for i) any overall results reported (if trials reported trial results at all).

We have also included a definition for counting the number of subgroups reported. Page6, para 3:

'One subgroup' indicates whether there are multiple levels (e.g., 'age' includes both <65 and >65-year-olds, 'sex' includes both women and men), we count each subgroup only once.

4) I like the motivation and the discussion with the reference to meta-analysis and the dilemma between "over-interpretation" and "completeness". I also like the suggestion of establishing common subgroups that should be reported and of providing results in digital repositories. But there are two aspects that I miss in the discussion (and also in the results). While it is mentioned that age was one of the most common variables for subgroup definition, the cut-off values used for definition of subgroups are not mentioned in the manuscript. In order to use age subgroups in a meta-analysis, the same (or at least similar) age subgroups had to be considered in the analysis of clinical trials. A second aspect that, in my opinion, should be discussed in more detail is the increased risk of false-positive findings due to multiplicity when more subgroups are investigated for the matter of completeness (which might include subgroups where not biological plausibility for treatment effect heterogeneity exists).

Thanks for these valuable comments. We acknowledge the importance the cut-off values for age and more complex work required and have added more details. Page 11, para 3:

"This would of course require an agreement as to what should constitute such a wider common set of subgroup effects (e.g., consistent definition of subgroups, identification of important subgroups across different diseases, establishment of cut-off values for continuous subgroups especially for age, or model continuous variables as continuous variables and account for non-linearity by fractional polynomials or cubic splines)."

We acknowledge the false positives due to multiple testing. We added the discussion for individual participant meta-analysis based on reviewer 1' comment (Page 11, para 4) and also mentioned the false positive issues.

"Another way to improve subgroup analysis is through individual participant data meta-analysis (IPD-MA), considered as the gold standard for exploring subgroup effects(8). Patients with specific or combinations of characteristics can be identified through IPD across different studies, then combined in a MA. It offers increased power compared to individual studies(9), allows for better flexibility to standardise subgroup definitions, and provides a higher credibility for findings compared to traditional MA(8, 10). However, it suffers from some disadvantages such as requiring substantial resources to obtain IPD, clean and create consistent data format across studies, data quality issues, and it has not been widely adopted (9, 11). Additionally, some frequently used regression-based methods in IPD-MA suffer from false positives(8). There is a trade-off between facilitating consistent subgroup reporting that would allow better meta-analysis of subgroups versus the increase in subgroup reporting which, if interpreted at the individual trial level, may lead to more false positives. Explicit guidance, reporting frameworks for subgroups should be developed to prevent misinterpretation and ensure the reliability of subgroup findings."

I have some additional minor comments:
- It is mentioned that this "was last updated in 04/2019". Does this refer to the search for publications

of the trials? That would have been quite a long time ago. - I think Figure 3 is a bit hard to read and interpret. The figure caption should provide more details or the figure should be adapted. The text refers to the "diagonals", but as the entries on the x-axis and on the y-axis are not identical, so there is no real diagonal.

Yes publications were updated in 04/2019. Please refer to our response to question 4 for reviewer 1 on page 3.

For Figure 3, we agree and have added red borders to the boxes where the subgroup is in the same category as the index condition and dropped any reference to diagonals.

- In the discussion, the following sentence can be found: "Alternatively, our null association for industry funding could be due to heterogeneity according to statistical significance of the primary outcome." I do not really understand what is meant here. Could you please explain and/or rephrase the sentence?

- I believe the terms "sponsor" and "funder" are used interchangeably but should be distinguished.

We agree that this sentence is confusing and have removed it instead stating that "our null association for industry funding could be due to the unmeasured confounding – ie unmeasured differences between industry and non-industry trials which are related to subgroup reporting".

Thanks. We acknowledge the difference between "sponsor" and "funder", and the terms mentioned in our paper mean sponsor. "Funder" terms have been changed to "sponsor", for example, "industry-funded" has been changed to "industry-sponsored."

.

- Labels in figures are generally too small.

Thank-you. We have increased the size of the figure labels.

References:

1. Murad MH, Wang Z. Guidelines for reporting meta-epidemiological methodology research. Evidence-based medicine. 2017;22(4):139-42.

2. Hanlon P, Hannigan L, Rodriguez-Perez J, Fischbacher C, Welton NJ, Dias S, et al. Representation of people with comorbidity and multimorbidity in clinical trials of novel drug therapies: an individual-level participant data analysis. Bmc Medicine. 2019;17(1).
3. Noncommunicable diseases [Available from: https://www.who.int/news-room/fact-sheets/detail/noncommunicable-diseases.
4. Taji Heravi A, Gryaznov D, Schandelmaier S, Kasenda B, Briel M, Adherence SR. Evaluation of Planned Subgroup Analysis in Protocols of Randomized Clinical Trials. Jama Network Open. 2021;4(10).
5. Wallace E, Salisbury C, Guthrie B, Lewis C, Fahey T, Smith SM. Managing patients with multimorbidity in primary care. Bmj-British Medical Journal. 2015;350.
6. Bennett SDD, Shafran R. Adaptation, personalization and capacity in mental health treatments: a balancing act? Current Opinion in Psychiatry. 2023;36(1):28-33.
7. Bursztyn M. Pioglitazone after Ischemic Stroke or Transient Ischemic Attack. New England Journal of Medicine. 2016;375(7):702-3.
8. Belias M, Rovers MM, Reitsma JB, Debray TPA, IntHout J. Statistical approaches to identify subgroups in meta-analysis of individual participant data: a simulation study. Bmc Medical Research Methodology. 2019;19(1).
9. Riley RD, Lambert PC, Abo-Zaid G. Meta-analysis of individual participant data: rationale, conduct, and reporting. Bmj-British Medical Journal. 2010;340.
10. Stewart LA, Tierney JF. To IPD or not to IPD? Advantages and disadvantages of systematic reviews using individual patient data. Evaluation & the Health Professions. 2002;25(1):76-97.
11. Simmonds M, Stewart G, Stewart L. A decade of individual participant data meta-analyses: A review of current practice. Contemporary Clinical Trials. 2015;45:76-83.

VERSION 2 – REVIEW

**REVIEWER**	Schoenenberger, Christof Manuel<br>Universitatsspital Basel, Division of clinical Epidemiology
**REVIEW RETURNED**	24-Mar-2024

**GENERAL COMMENTS**	I think the manuscript has substantially improved with this round of revisions. I would like to point out some remaining minor comments.<br><br>1) I encourage to report any used methods in the method section and not in the result section since it makes it more complicated to read. For example, move the description of the moved models to the method section in this paragraph: Figure 2 shows associations for any overall result reporting (yes/no among 2,235 trials), any subgroup reporting (yes/no among 1,082 trials reporting overall results), and total number of subgroups reported (among those trials reporting subgroup), using logistic regression models and a Poisson model, respectively.<br>2) Where is figure 1? I think you start counting at figure 2?<br>3) Figure 2:<br>a. What is the difference between stroke and cerebral infarction? Why is there DM type 1, DM type 2 and DM? And the differentiation of angina pectoris and acute coronary syndrome is a bit strange. Overall, I think you should have a look again on this classification.<br>b. Would be great if you could add absolute numbers and a short description of the figure directly to the figure.<br>c. Thank you for the overall simplification of the figure.

	4) I still find figure 3 unhelpful. It shows a fairly obvious information in a very complex graphic. The red boxes don't make it any better in my opinion. I would remove this figure from the manuscript and list it as an appendix.<br>5) I think the video for the shiny app is very helpful. Nice solution.

**REVIEWER**	Haller, Bernhard<br>Technische Universität München
**REVIEW RETURNED**	11-Mar-2024

**GENERAL COMMENTS**	I reviewed the revised version of the manuscript and I believe that it has relevantly improved. The video instruction for the heat map was very helpful. Still, I have some minor issues that should be considered.<br><br>For some of the changes that were made, the wording should be checked as the sentences now contain mistakes (e.g. "... that the coefficient corresponds is to a 10-fold ..." or "For the quasi-Poisson model, ... and covariates included were the same as regression models."<br><br>The sentence "These variables were mutually adjusted." should be deleted, as it is not proper for the description of a multivariable model.<br><br>I think, Figure 3 and the red borders should be explained in more detail. It was hard to understand for me when I read the section for the first time.<br><br>I like the section on individual patient data meta-analysis that was added based on another reviewer's comment. I think a sentence on limitations of the approach due to data protection and confidentiality issues should be included.

VERSION 2 – AUTHOR RESPONSE

Formatting Amendments (where applicable):

Reviewer: 2

Dr. Bernhard Haller, Technische Universität München

I reviewed the revised version of the manuscript and I believe that it has relevantly improved. The video instruction for the heat map was very helpful. Still, I have some minor issues that should be considered.

For some of the changes that were made, the wording should be checked as the sentences now contain mistakes (e.g. "... that the coefficient corresponds is to a 10-fold ..." or "For the quasi-Poisson model, ... and covariates included were the same as regression models."

Thanks. We have checked and corrected them.

*"*log-transformed with a base of 10, so that the coefficient corresponds to the increase in overall results reporting or subgroup reporting per 10-fold increment in sample size…*"*

For the quasi-Poisson model, the outcome was the count of subgroups per trial, and covariates included were the same as those in the regression models.

The sentence "These variables were mutually adjusted." should be deleted, as it is not proper for the description of a multivariable model.

Thanks. It has been deleted.

I think, Figure 3 and the red borders should be explained in more detail. It was hard to understand for me when I read the section for the first time.

Thanks. More details have been added to make it clearer.

Page 9, para 1:

"Where the index condition and subgroup pertain to the same organ system, the cells are outlined in red. Otherwise, if they are in different organ systems, the cells are not bordered. Frequencies above 5% were generally seen on the cells with red borders (e.g., 13% CVD trials reported a non-index condition CVD subgroup - e.g., stroke trials reported hypertension as a subgroup which are both CVDs). Where there were high percentages not in red borders, the subgroup conditions were either known causes or known sequelae of the index condition such as…"

Figure 3. Comorbidity subgroup reporting.

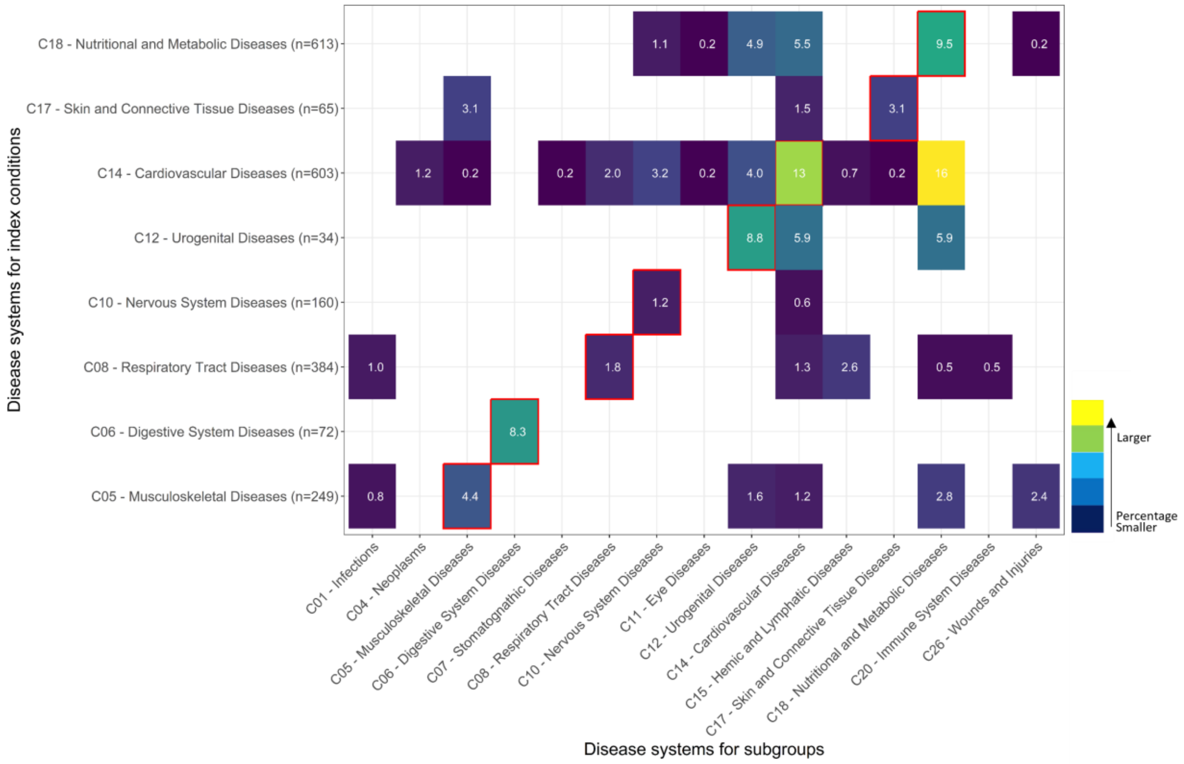

I like the section on individual patient data meta-analysis that was added based on another reviewer's comment. I think a sentence on limitations of the approach due to data protection and confidentiality issues should be included.

Thanks for this suggestion. It has been added in the page 12, para 2:

*"*Moreover, there are legal and ethical considerations regarding privacy and confidentiality when sharing IPD (3). Thus there are also challenges in accessing and using IPD to examine subgroup effects.*"*

Reviewer: 1

Dr. Christof Manuel Schoenenberger, Universitatsspital Basel

I think the manuscript has substantially improved with this round of revisions. I would like to point out some remaining minor comments.

1) I encourage to report any used methods in the method section and not in the result section since it makes it more complicated to read. For example, move the description of the moved models to the method section in this paragraph: Figure 2 shows associations for any overall result reporting (yes/no among 2,235 trials), any subgroup reporting (yes/no among 1,082 trials reporting overall results), and total number of subgroups reported (among those trials reporting subgroup), using logistic regression models and a Poisson model, respectively.

Thanks. We have cut down the method part as below. Figure 2 shows the results, which we think should remain in the results section.

“Figure 2 shows associations for any overall result reporting (yes/no among 2,235 trials) and any subgroup reporting (yes/no among 1,082 trials reporting overall results).”

2) Where is figure 1? I think you start counting at figure 2?

Figure 1 is the Screening of subgroup analyses from papers reporting any overall trial results showed below.

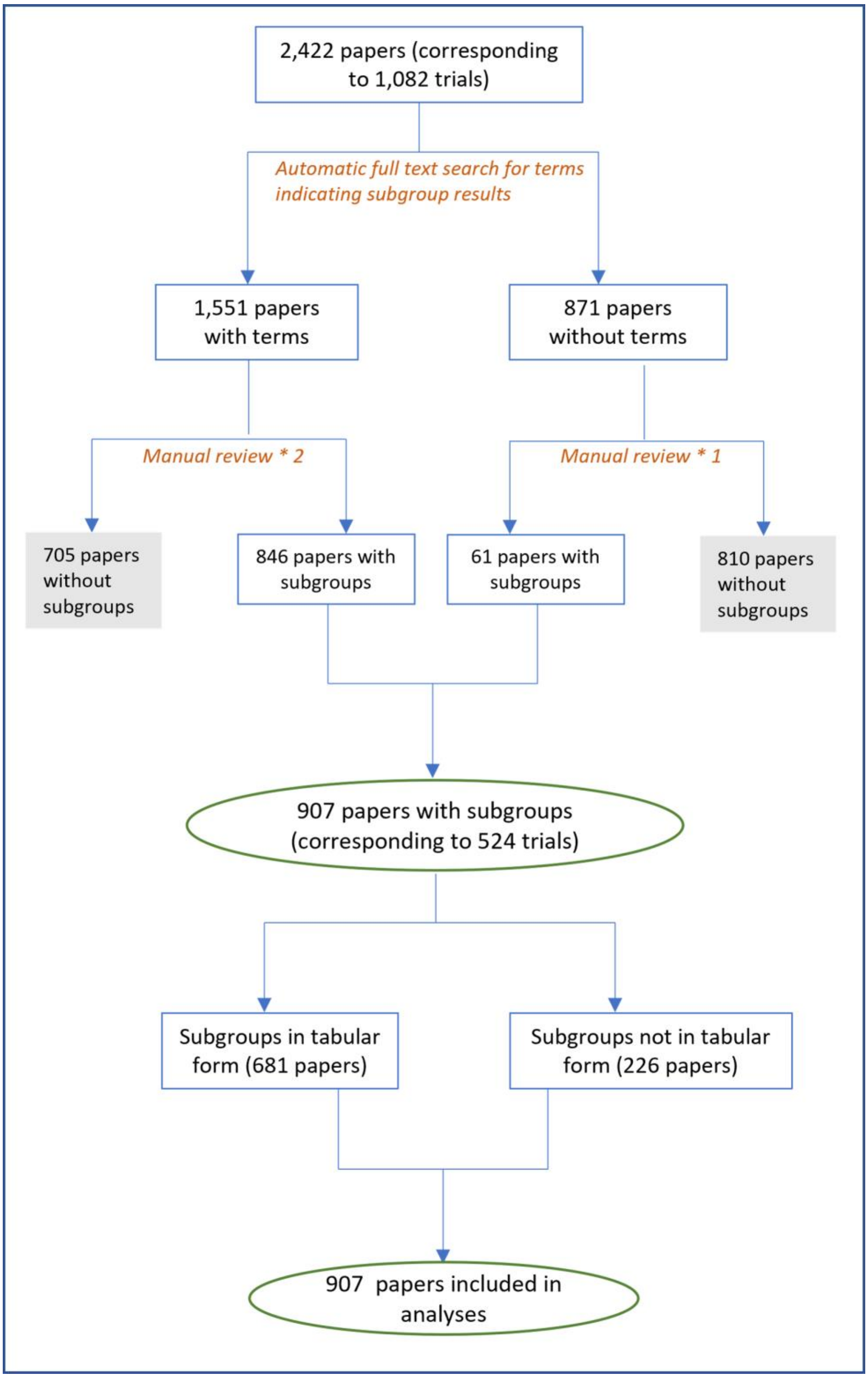
2,422 papers (corresponding to 1,082 trials)
Automatic full text search for terms indicating subgroup results
1,551 papers with terms
871 papers without terms
Manual review * 2
Manual review * 1
705 papers without subgroups
846 papers with subgroups
61 papers with subgroups
810 papers without subgroups
907 papers with subgroups (corresponding to 524 trials)
Subgroups in tabular form (681 papers)
Subgroups not in tabular form (226 papers)
907 papers included in analyses

3) Figure 2:

a. What is the difference between stroke and cerebral infarction? Why is there DM type 1, DM type 2 and DM? And the differentiation of angina pectoris and acute coronary syndrome is a bit strange. Overall, I think you should have a look again on this classification.

We apologise for the lack of clarity. We should have stated that the reported index conditions were not assigned by us, but by the trial sponsors and clinicaltrials.gov based on MeSH terminology. Thus, where MeSH distinguishes between terms (such as in the case of cerebral infarction and stroke – please see https://meshb.nlm.nih.gov/record/ui?ui=D002544 *and* https://meshb.nlm.nih.gov/record/ui?ui=D020521 *for comparison of the concepts for both terms*), rather than collapse these to form our categories, we have retained the original MeSH assigments*. Please see this link for examples of the use of the terms in the original trial registrations (*https://clinicaltrials.gov/search?term=NCT00200356%20OR%20NCT00272090%20OR%20NCT00027066%20OR%20NCT00202566%20OR%20NCT00134147%20OR%20NCT00251576%20OR%20NCT00082407*.*

We have now added the following text to explain the choice of terms we used for index conditions.

Page 6, para 2:

*For index conditions, we used the original MeSH terms assigned when trials were registered. Briefly, when registering a study, data submitters are required to provide condition using MeSH terms. Furthermore, an NLM algorithm assesses submitted text and assigns MeSH terms. More details of this process are available in section 2.1 "*Use of MeSH Terminology in the ClinicalTrials.gov Database*" (4).*

b. Would be great if you could add absolute numbers and a short description of the figure directly to the figure.

Thanks. They have been added.

Figure 2. The associations between a) trial characteristics, b) chronic diseases, and subgroup reporting, and overall results reporting, respectively.

2a) Trial characteristics

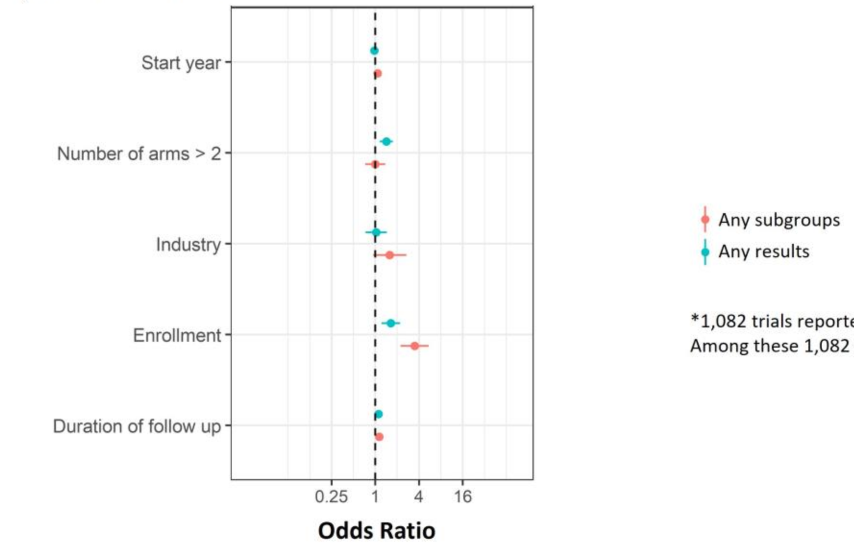

*1,082 trials reported overall results out of 2,235 trials. Among these 1,082 trials, 524 reported subgroups.

2b) Chronic diseases

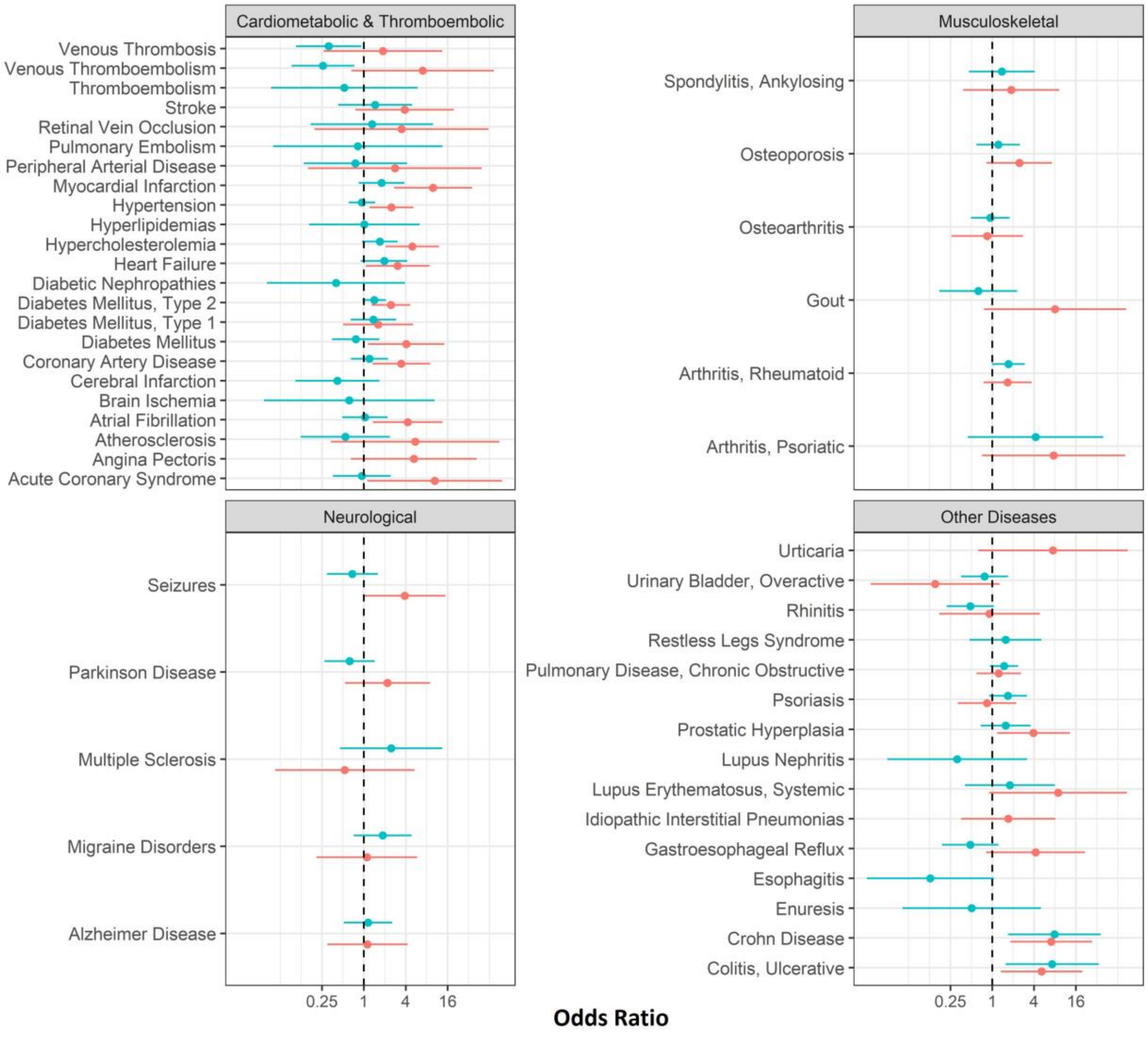

c. Thank you for the overall simplification of the figure.

4) I still find figure 3 unhelpful. It shows a fairly obvious information in a very complex graphic. The red boxes don't make it any better in my opinion. I would remove this figure from the manuscript and list it as an appendix.

Thanks. However, while we appreciate the helpful comments about this manuscript, we feel that this figure does communicate an interesting finding about what sort of comorbid conditions trials tend to report on. We would therefore be keen to retain it. Nonetheless, if the editorial committee would like us to remove this figure we would of course be happy to do so.

5) I think the video for the shiny app is very helpful. Nice solution.

Thank-you for the previous feedback about the heatmap. We are pleased that recording a short video helped address this deficiency.

Reviewer: 2
Competing interests of Reviewer: None

Reviewer: 1
Competing interests of Reviewer: No competing interests to declare

References:

1. Lees JS, Hanlon P, Butterly EW, Wild SH, Mair FS, Taylor RS, et al. Effect of age, sex, and morbidity count on trial attrition: meta-analysis of individual participant level data from phase 3/4 industry funded clinical trials. Bmj Medicine. 2022;1(1).
2. Butterly E, Hanlon P, Shah ASVH, Hannigan L, McIntosh ES, Lewsey JM, et al. Comorbidity and health-related quality of life in people with a chronic medical condition in randomised clinical trials: An individual participant data meta-analysis. Plos Medicine. 2023;20(1).
3. Mello MM, Lieou V, Goodman SN. Clinical Trial Participants' Views of the Risks and Benefits of Data Sharing. New England Journal of Medicine. 2018;378(23):2202-11.
4. Tasneem A, Aberle L, Ananth H, Chakraborty S, Chiswell K, McCourt BJ, et al. The Database for Aggregate Analysis of ClinicalTrials.gov (AACT) and Subsequent Regrouping by Clinical Specialty. Plos One. 2012;7(3).
